# The transcriptome from asexual to sexual in vitro development of *Cystoisospora suis* (Apicomplexa: Coccidia)

**DOI:** 10.1038/s41598-022-09714-8

**Published:** 2022-04-08

**Authors:** Teresa Cruz-Bustos, Anna Sophia Feix, Manolis Lyrakis, Marlies Dolezal, Bärbel Ruttkowski, Anja Joachim

**Affiliations:** 1 Institute of Parasitology, Department of Pathobiology, University of Veterinary Medicine Vienna, Veterinärplatz 1, 1210 Vienna, Austria; 2 Platform for Bioinformatics and Biostatistics, Department of Biomedical Sciences, University of Veterinary Medicine Vienna, Veterinärplatz 1, 1210 Vienna, Austria

**Keywords:** Cell biology, Genetics, Microbiology, Diseases

## Abstract

The apicomplexan parasite *Cystoisospora suis* is an enteropathogen of suckling piglets with woldwide distribution. As with all coccidian parasites, its lifecycle is characterized by asexual multiplication followed by sexual development with two morphologically distinct cell types that presumably fuse to form a zygote from which the oocyst arises. However, knowledge of the sexual development of *C. suis* is still limited. To complement previous in vitro studies, we analysed transcriptional profiles at three different time points of development (corresponding to asexual, immature and mature sexual stages) in vitro via RNASeq*.* Overall, transcription of genes encoding proteins with important roles in gametes biology, oocyst wall biosynthesis, DNA replication and axonema formation as well as proteins with important roles in merozoite biology was identified. A homologue of an oocyst wall tyrosine rich protein of *Toxoplasma gondii* was expressed in macrogametes and oocysts of *C. suis*. We evaluated inhibition of sexual development in a host-free culture for *C. suis* by antiserum specific to this protein to evaluate whether it could be exploited as a candidate for control strategies against *C. suis*. Based on these data, targets can be defined for future strategies to interrupt parasite transmission during sexual development.

## Introduction

*Cystoisospora suis* (syn. *Isospora suis*^1^) is a protozoan parasite of the phylum Apicomplexa (Class Conoidasida, subclass Coccidiasina, order Eucoccidiorida, family Sarcocystidae). This phylum contains almost exclusively obligate endoparasites of animals, including species of great medical and veterinary relevance such as *Plasmodium* spp., *Eimeria* spp., *Cryptosporidium* spp., and *Toxoplasma gondii*^2^. *Cystoisospora suis* is the causative agent of neonatal porcine cystoisosporosis (coccidiosis). The disease is characterized by generally self-limiting diarrhea and reduced weight gain in suckling piglets, mostly in the first three weeks of life, and leads to unthriftiness at weaning, considerably impairing animal health and productivity^3,4^. It has a worldwide distribution, and infections are very common, particularly in young animals^5^.

The life-cycle of *C. suis* consists of asexual multiplication (sporogony, merogony) followed by sexual development (gamogony) with the production of gamonts (syn. gametocytes). Merogony and gamogony take place entirely in one host and the sporogony in the environment. After ingestion of sporulated oocysts, invasive stages are released and infect the gut epithelial cells to reproduce asexually within an intracellular vacuole^6^. The final generation of merozoites is considered to be sexually committed as the first step towards sexual development. The early gamonts, already differentiated into spherical micro- and macrogamonts (syn. micro- and macrogametocytes), are both immobile and morphologically very similar in shape and size^7^. Gamonts develop further into clearly differentiated macro- and microgametes. The life cycle eventually proceeds with the fertilization by fusion of a motile flagellated microgamete with a large and immobile macrogamete, leading to the formation of a diploid zygote. After fertilization, the immature oocysts are excreted with the feces and undergo sporogony in the environment. Several divisions of the zygote by meiosis and mitosis result in infectious haploid sporozoites contained in the mature oocyst^8,9^. Previous studies have shown that the development of *C. suis *in vitro through the entire lifecycle is comparable with the lifecycle *in vivo*^10^. This makes it possible to observe, harvest and examine sexual stages during the short time frame in which they occur. After in vitro merogony in epithelial host cells *C. suis* can also continue gamogony in a host cell-free environment, sugesting that gamete production and fusion occurs extracellularly^11^. The complete life cycle of *C. suis* in a cell line representing the natural host cell type and species provides a unique model among coccidian parasites and can be used to address a wide range of topics (RNAseq, proteomics), especially with regard to the sexual development of coccidia.

Comparative RNA-seq analysis can be exploited to uncover molecules and pathways critical to parasite biology. In recent years, transcriptomic and proteomic analyses carried out in Apicomplexa revealed different genes coding for proteins related to the sexual development and unravelled key components common to it in different species. Based on profiling quantitative changes in gene transcription, stage-specific genes have been identified in oocysts, sporozoites, second and third-generation merozoites and gametocytes of *E. tenella*, *E. maxima* and *E. acervulina*^12–17^, in tachyzoites, bradyzoites, sporozoites and oocysts of *T. gondii*^18–24^, in tachyzoites of *Neospora caninum*^25^, in tachyzoites of *Besnoitia besnoiti*^26^, oocysts, sporozoites and intracellular stages of *Cryptosporidium parvum*^27^, and in human and mosquito stages^28–30^ and gametocytes of *Plasmodium falciparum* and *P. vivax*^31–33^.

According to recent reevaluations of the coccidian phylogeny, the position of *C. suis* in the family Sarcocystidae constitutes an outgroup of the cluster containing the genera *Neospora*, *Hammondia* and *Toxoplasma*^34,35^. The transcriptional profile of multiplying asexual stages (tachyzoites) of *T. gondii,* the closest relative to *C. suis*, revealed an upregulation in genes encoding proteins involved in host cell adhesion and invasion, intracellular development and multiplication, resistance to host stress, gliding motion and ribosomal proteins in comparison to resting stages (bradyzoites). The AP2 (ApiAP2) family was identified as a major class of transcriptional regulators that are found across all Apicomplexa and modulate key regulatory decisions of parasite development^23^. Only a small number of asexual stages differentiates into gametes, and therefore this step is considered a bottleneck of development^36^, and it can be assumed that this is relevant for all Apicomplexa that produce gametocytes^37^. Proteins with important roles in sexual development have been described, including members involved in macrogamete development, oocyst wall formation, glycosilation and proteolytic cleavage of the oocyst wall proteins^38,39^, axoneme and flagella assembly and construction, DNA replication, microgamete budding from microgamonts and gamete fusion^40,41^.

In the present study we performed RNA-seq of *C. suis* harvested at different points during development – specifically, asexual stages (merozoites) as a baseline, and immature and mature sexual stages (microgametes, macrogametes and early oocysts) to provide a better understanding of the developmental process and regulation of sex differentiation of *C. suis *in vitro.

## Results and discussion

### Overview of RNA sequencing of *C. suis* merozoites and sexual stages

Transcriptome sequencing was carried out at three time points characterizing three different steps in the development of *C. suis* to determine the transcript levels. Epithelial cells were infected with freshly excysted sporozoites and culture supernatants containing developed parasite stages were harvested at different time points: time points T1 (days 6–8 after infection) for the merozoites, T2 (days 9–11), containing merozoites and immature sexual stages, and T3 (days 12–14), containing mainly mature sexual stages and oocysts. Total RNA was extracted from seven biological replicates for each time point, DNase-treated and quality assessed by automated gel electrophoresis. Parasite-specific large ribosomal RNA bands (26S and 18S) were detected in all samples. Although contamination with host RNA (28S) was also observed, this was not a strong concern since we performed read mapping to the *C. suis* genome. Approximately 21 million reads were generated for each sample. Data were mapped to the combined genomes of *C. suis* (strain Wien I) and the pig host, *Sus scrofa* (Sscrofa11.1). After filtering out *S. scrofa*, at least 21% of the mapped reads of each replicate were assigned to the *C. suis* genome, this provided a robust data for quantitative analysis of gene transcript levels (for details of the total number of reads per replicate, see Table S1).

### Identification of differentially expressed genes

**Figure 1:**
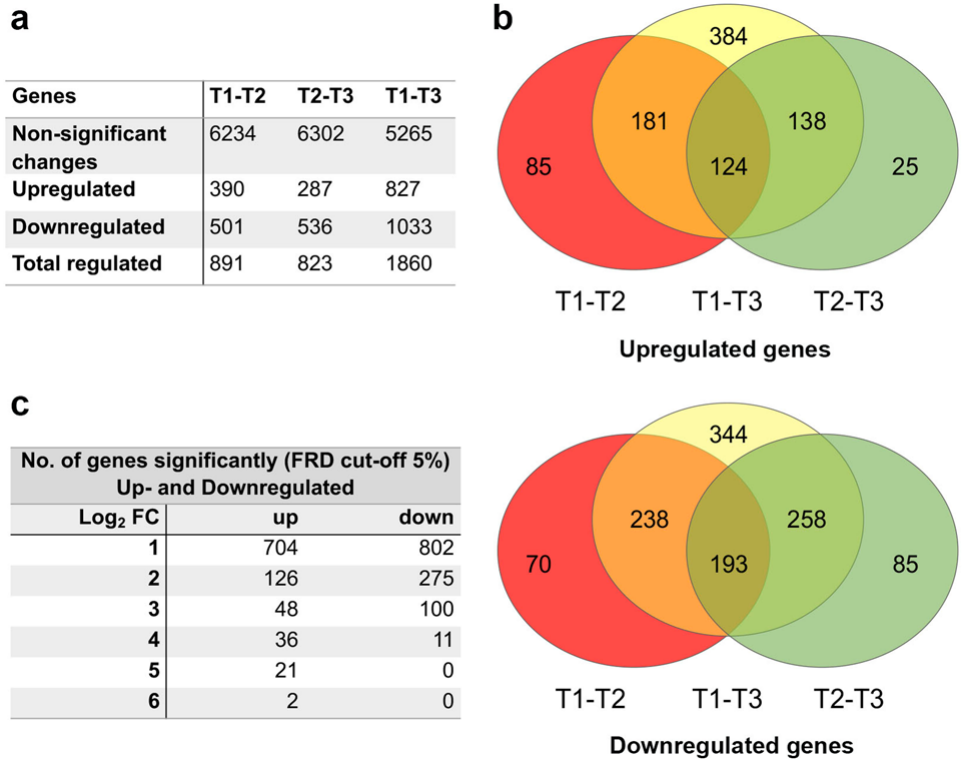
Identification of upregulated and downregulated genes in sexual stages using differential expression analysis. (**a**) Summary of the differential expression analysis of early sexual stages compared to merozoites (T1-T2), late sexual stages (T1-T3) and late sexual stages compared to early sexual stages (T2-T3) showing the number of genes up or down-regulated of all predicted *C. suis*. (**b**) Venn diagrams showing the overlap between the genes that were up- and down-regulated in early and late sexual stages compared with asexual stages. A total of 443 upregulated and 689 downregulated genes were identified in this overlapping region. (**c**) Summary of total number of genes (937 upregulated and 1188 downregulated) in early and late sexual stages.

In order to identify upregulated or downregulated transcripts, quantified RNASeq mapping was used to generate quantitative profiles for individual differentially expressed genes (DEG) between developmental stages of *C. suis* harvested at different time points. Lowly expressed *C. suis* genes (4,418 out of 11,543) were excluded from subsequent analysis (filtering criteria: cpm > 4 and count > 30 for at least four replicates at each time point). The remaining 7,125 genes were tested for differential gene expression with a mixed model accounting for the repeated measures structure of our data. At a False Discovery Rate (FDR) cut-off of 5% and a minimum absolute log2 fold change of 1, we found 891 and 1,860 differentially expressed genes at T2 and T3 compared to T1, and 823 genes differentially expressed at T3 compared to T2, respectively (Fig. 1a).

The primary goal of this study was the identification of genes with elevated expression levels at sexual stages to identify genes and proteins that may be related to this last step of development of the Coccidia, including *C. suis*. In total, 937 upregulated and 1188 downregulated genes were identified in sexual stages compared to T1-T2 (Fig. 1b and c), representing 8.11% respectively 12.53% of all predicted *C. suis* genes. The gene identification, description and transcript abundance levels for each of these genes, at each developmental stage, are provided in Table S2.

### qRT-PCR validation

**Figure 2:**
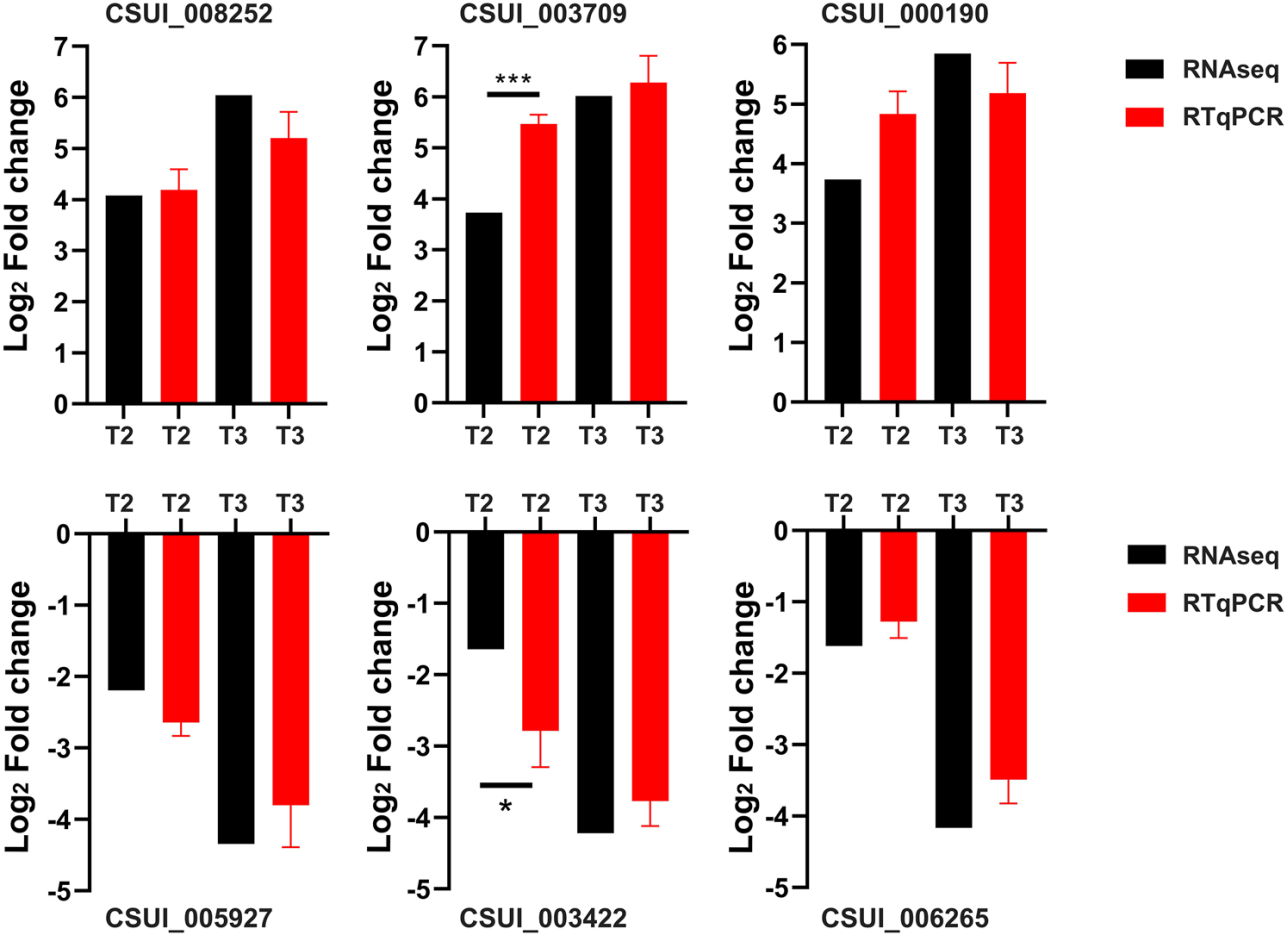
Verification of the gene expression profiles by qRT-PCR. Six genes were selected randomly for validation of the RNA-seq data. According to the RNA-seq results, the expression levels of CSUI_008252, CSUI_003709, CSUI_000190 were upregulated at T2 and T3, and the expression levels of CSUI_005927, CSUI_003422 and CSUI_006265 were downregulated at T2 and T3. Glyceraldehyde-3-phosphate and actin were used for normalization. Values represent the mean ± standard deviation (SD). Asterisks represent significant difference (*P < 0.05, **P < 0.01***, *P* < 0.001, ****P < 0.0001).

The gene expression profile identified by RNAseq was validated by selecting six different genes for qRT-PCR analysis with specific primer sequences. The transcripts levels were calculated according to the 2-ΔΔCt values^112^ (see amplication efficacies for primers in Supplemental file S1). Using glyceraldehyde-3-phosphate (GAPDH) and actin as a reference genes, expression levels determined by qRT-PCR were consistent with those obtained by RNA-seq (Fig. 2), confirming the accuracy and reliability of the RNA-seq results. Thus, the data generated here can be used to investigate stage-specific expression of genes that show different expression levels among different developmental stages.

### Gene ontology classification

**Figure 3:**
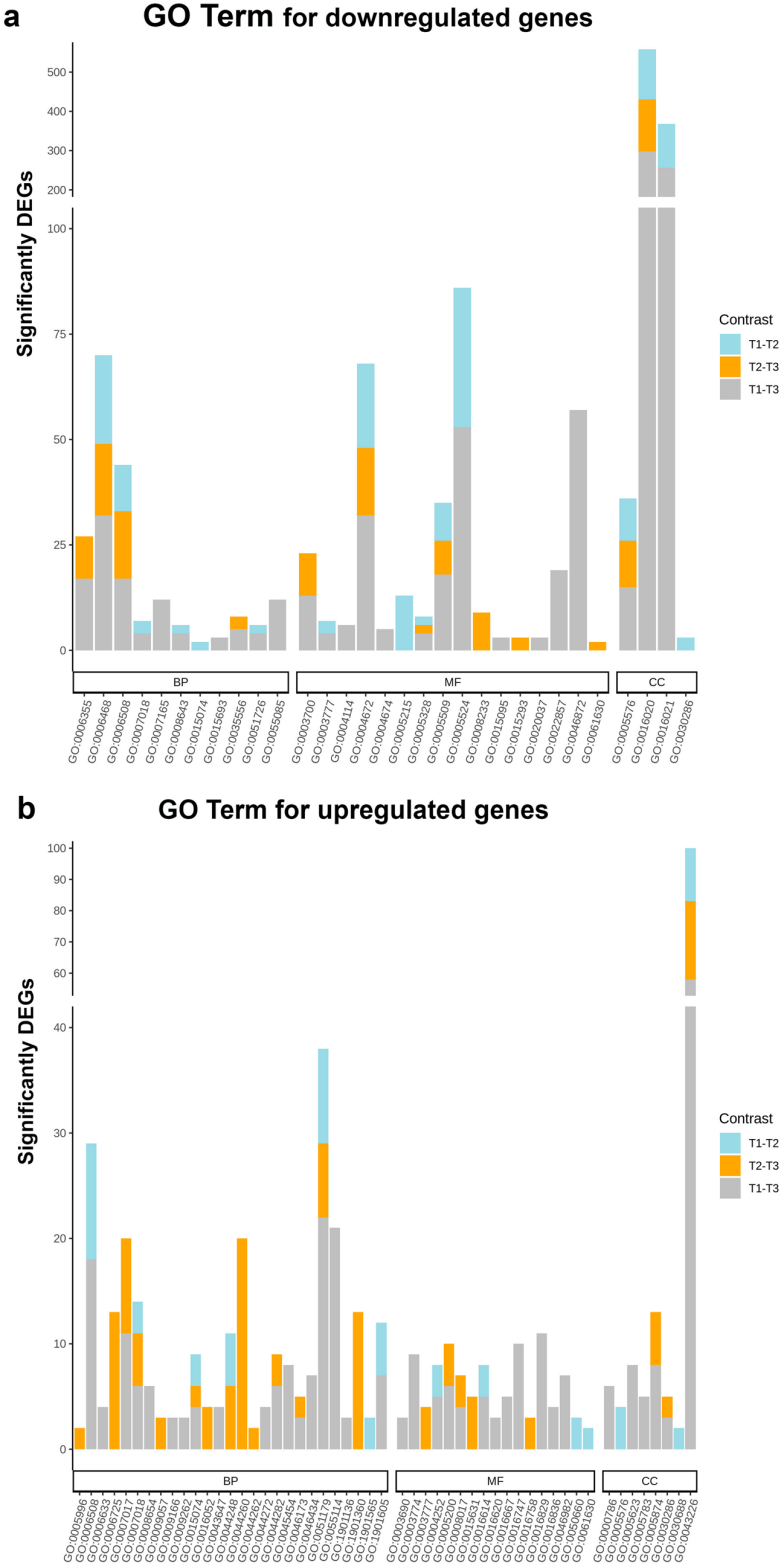
Gene ontology (GO) analysis of differently expressed genes. Differentially expressed genes (DEGs) are classified into three main categories: biological process (BP), molecular function (MF) and cellular component (CC). The identified functions for each corresponding numbers GO category are shown in supplemental file 2.

Gene ontology enrichment analysis was conducted by Fisher's exact test taking into account the GO hierarchy. At a significance cut-off of 5%, 27 biological processes, 17 molecular functions and eight cellular components were significantly enriched in upregulated genes. The top five GO terms enriched in upregulated genes were associated with localization, proteolysis, oxidation–reduction process, microtubule-based movement and cellular catabolic processes which together support subsequent gamogony, fertilization and oocyst wall formation. For the downregulated genes, 11 biological processes, 16 molecular functions and 4 cellular components were significantly enriched. The top five GO terms enriched in downregulated genes were protein phosphorylation, proteolysis, regulation of transcription, signal transduction and transmembrane transport which are implicated in host invasion, merozoite reproduction and gamogony as well as and parasite-host immunological interactions (Fig. 3).

### Transcripts down- or upregulated in sexual stages

**Figure 4:**
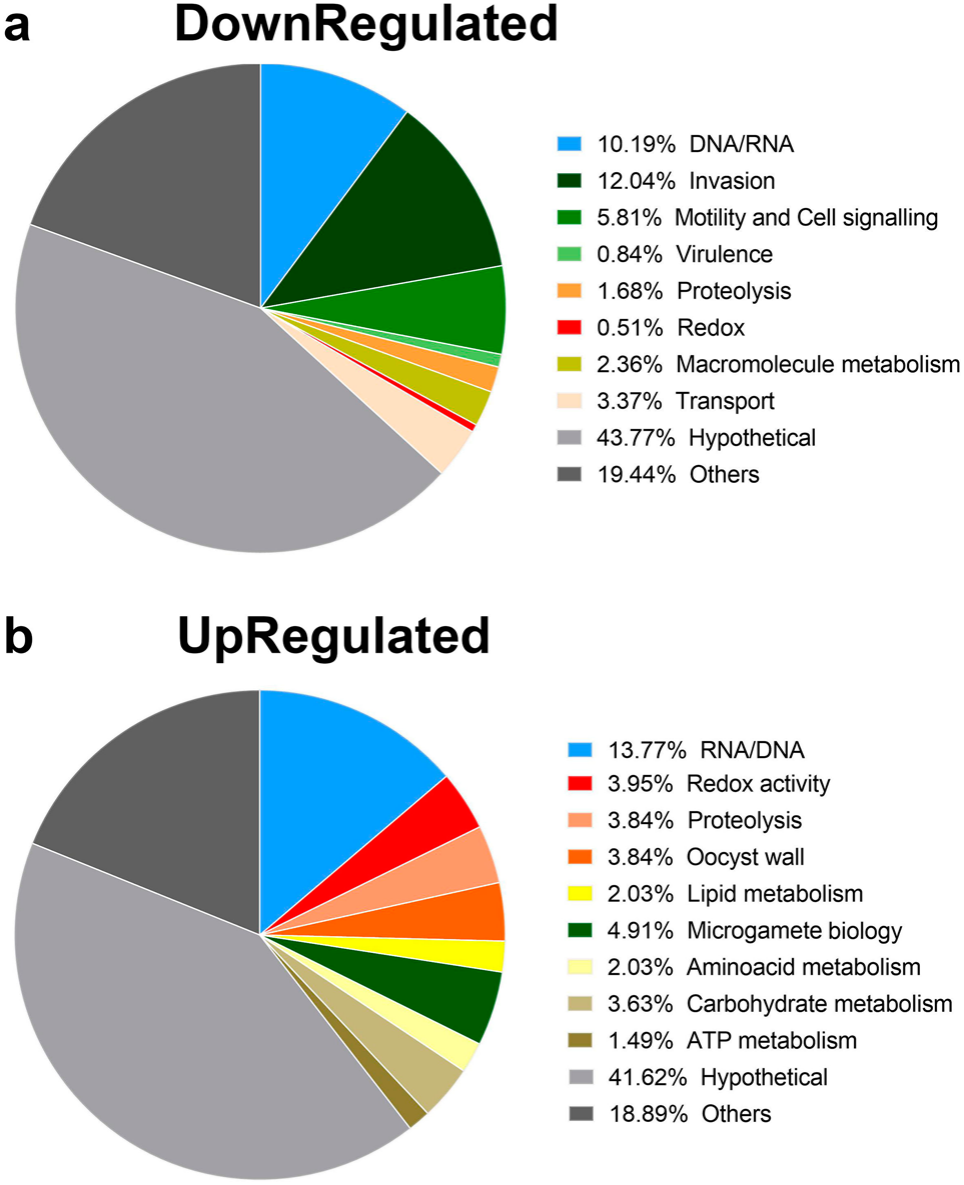
Biological functions of proteins coded by downregulated** (a)** and upregulated** (b) **genes. The biological functions of proteins coded by each of the 1188 down- and 937 upregulated transcripts was assigned manually based on ToxoDB®, Blast2Go® or previously published annotations. The pie charts represent the relative proportion of these different biological functions within up- and down regulated genes.

Within the subset of down- and upregulated transcripts identified in *C. suis*, a large proportion coded for hypothetical proteins (Fig. 4a and b)—an expected observation, given the still limited understanding of coccidian sexual biology^14,41,42^. As expected, genes coding for previously characterised merozoite proteins with putative roles in host-cell attachment and invasion, motility, signaling, virulence and transport were most distinctly downregulated (Fig. 4a); detailed information on these is given in Sect. Transcripts down- or upregulated in sexual stages. Other putative functions (not discussed in detail) included proteolysis, redox activity and DNA/RNA related proteins. Genes coding for previously characterised gametocyte antigens and oocyst wall proteins are among the most highly transcribed gametocyte genes including proteins with putative roles in glycosylation, protease activity, redox activity and fatty acid metabolism, surface and oocyst wall formation, as well as components of microgamete flagella (Fig. 4b) and further details are given in  “Macrogamete and oocyst-specific genes” and “Microgamete-specific genes” sections. Other putative functions (not discussed in detail) included: (1) metabolism, including the energy metabolism, aminoacid synthesis and carbon source, and (2) DNA/RNA binding, which may play a role in gene regulation that is not yet further specified for coccidia. About 20% of the proteins found in both sets of regulated transcripts have diverse functions with undefined roles in parasite biology, *e.g.,* kinase activity, calcium and metal binding or membrane components.

### Identification of genes downregulated in sexual stages

Asexual stages of Coccidia develop strictly intracellularly, and have developed various strategies to ensure cell invasion and intracellular persistence. The invasive stages have specialized cellular structures and organelles attached to their membranes^43^. The apical polar complex is composed of secretory organelles (micronemes and rhoptries) and structural elements (conoid and polar rings). *T. gondii* secretes a broad spectrum of proteins to infiltrate its host cells and to regulate the expression of host proteins, including micronemal proteins (MICs) and PAN/Apple domains, rhoptry and rhoptry neck proteins (ROPs and RONs) and dense granules (GRAs)^44–47^. We detected 19 genes coding for nine different MICs and seven coding for PAN-domain containing proteins, 32 ROPs and RONs and two dense granule proteins downregulated in sexual stages (Table S3). Internalization of asexual stages is achieved by active participation of the parasite^48–50^. The process of gliding requires the coordinated secretion and translocation of proteins via the actin-based cytoskeleton. Parasites use the gliding motion to establish host cell adhesion to generate enough traction to drive themself into the host cell^51,52^. This initial contact is mediated by proteins released from the micronemes^44^. Of these, the best characterized are the Apical Membrane Antigen 1 (AMA1) and yet anonymous thrombospondin-related proteins which bind directly to the motor complex of the adhesion site^53–55^. We identified three genes related to these proteins that were downregulated in sexual compared to asexual stages.

Invasion, replication and egress require dynamic changes in the cellular architecture of the parasite. The inner membrane complex (IMC) is a structural element involved in these morphological changes. The IMC of *T. gondii* is a peripheral membrane system composed of flattened alveolar sacs (alveoli) underlying the plasma membrane, coupled to a supporting cytoskeletal network. The IMC plays major roles in parasite intracellular replication, motility and host cell invasion^56–58^. The best studied group of IMC proteins are components of the motor complex—also referred to as the “glideosome” – in *T. gondii*^59,60^. This actin-myosin motor complex powers the required cell motility, and the proteins identified include myosins, tubulins, actins and glideosome-associated proteins. Twenty-six genes coding for these proteins were identified in asexual stages of *C. suis*. Another interesting group of IMC proteins, such as the Inner Membrane Complex Protein 1 of *T. gondii* (TgIMC1)^56^ are the alveolins of which we identifed 14 genes in *C. suis*. Beside the alveolins, other additional IMC-associated peripheral membrane proteins like the IMC subcompartment-proteins (ISPs) were identified^61^. The specific signalling pathways which regulate the activity of the glideosome are still not known. Regulation of adhesin release from micronemes and glideosome activity are linked to environmental signals that ensure proper activation and suppression of gliding motility^49^. Extracellular K^+^ and cytosolic Ca_2_^+^ concentrations have been implicated in the activation of gliding motility^62^. A role for cyclic nucleotide signaling has been unveiled, and phosphorylation, and methylation events also regulate the gliding motility^63^. A total of 43 genes encoding proteins that are involved in phosphorylation and cell signalling, signal transduction and calcium regulation were identified in *C. suis* (Table S3).

The surface of *T. gondii* tachyzoites and bradyzoites is covered with glycosylphosphatidylinositol (GPI)-anchored antigens, most of which are members of the large family of surface antigen (SAG)-related (SRS) proteins which includes the SAG1-like and the SAG2-like sequence branches, SAG and SUSA (SAG-unrelated surface antigens). These proteins have diverse functions. Presumably they facilitate adhesion to and invasion of host cells, and play a role in immune evasion and defining host specificity^64–66^. In *C. suis*, 53 SAGs or SRSs were identified, and their downregulation indicates that sexual stages have less interactions with host cells and the host’s immune system, maybe because the do not reinvade cells and are rather short-lived, progressing quickly from gamonts to oocysts^11^.

We could show that genes encoding proteins that play an active role in the invasion of host cells are downregulated in the early and late sexual stages of *C. suis*, supporting the assumption that these stages do not invade host cells. These molecular clues consequently suggest that the fertilization process (and consequently oocyst formation) occurs extracellularly which facilitates and accelerates the discharge of oocysts into the enviroment – but at the same time makes these stages accesible to specific antibodies for immunological control.

### Genes involved in cell cycle regulation

During the progression from asexual to sexual stages in in vitro culture, we identified orthologues of 12 genes coding for proteins found specifically in bradyzoites of *T. gondii,* involved in tissue cyst wall formation^67,68^, an orthologue of the bradyzote antigen 1^21^, a Myb-like transcription factor^69^, two heat shock proteins and one serpin found in this stage (Table S4). The existence of bradyzoites as persisting intracellular stages has never been demonstrated in *C. suis,* neither *in vivo*^70^ nor in vitro*.* However, other species of the genus *Cystoisospora* can develop resting monozoic tissue cyst stages^71,72^. The role of these putative proteins in merozoite biology of *C. suis* remains to be investigated. The commitment of type II merozoites to sexual differentiation during the final phase of asexual development is a key process during the life cycle of Apicomplexan parasites^73^. DNA binding proteins (ApiAP2 factors) are related to the APETALA family of transcription factors which play key roles in the development and environmental stress response pathways of plants^74^. The ApiAP2 family was discovered in the genomes of various Apicomplexan species^75^. In *Plasmodium*, they play a role in stage conversion^76^ and are related to sexual commitment of blood-stages^77–79^. Currently, 67 ApiAP2 domain-containing proteins are annotated in the *Toxoplasma* genome, with 24 being expressed cyclically during the tachyzoite division cycle^18,80^ and six in bradyzoite development^81^. The genome of *C. suis* encodes 64 AP2 factors of which we found 23 AP2 factors downregulated and 7 AP2 factors upregulated in the sexual stages, possibly linked to the observed stage conversion from type II merozoites to gamonts and onwards to gametes.

### Macrogamete and oocyst-specific genes

The wall composition of coccidian oocysts has previously been characterized in great detail^39^. Oocyst wall proteins (OWP) and gametocyte-specific proteins (GAM)-proteins were previously characterized in *Eimeria*, *Toxoplasma* and *Cryptosporidium* as the main protein constituents of the oocyst wall^38,82–86^. In *C. suis*, 12 transcripts, originally described in oocysts of *T. gondii* and *E. maxima,* were confirmed to be upregulated at T2 and T3 (Tables 1 and S5). The putative oocyst wall proteins encoded by CSUI_008806 and CSUI_006207 showed homology to TgOWP6 and TgOWP1, respectively, cysteine-rich oocyst wall proteins of the wall-forming bodies with a vital role in oocyst wall formation^87^. Seven genes that encode proteins of novel OWP candidates were not highly homologous with established OWPs although all of them share characteristic cysteine repeats^86^. The oocyst and sporocyst walls of *C. suis* display autofluorescence under 405 nm laser light, presumably due to the dityrosine bonds formed between tyrosine-rich proteins in the oocyst wall^88^. Three hypothetical proteins which are tyrosine-rich (> 14% and 7% of tyrosine) are homologous to genes identified in the oocyst proteome of *T. gondii* and the sporocyst wall of *E. tenella*^20,89,90^. Recent studies on glycosylation in *Toxoplasma* demonstrated its importance for cyst wall rigidity and parasite persistence in the environment^91^. In *Eimeria*, glycoproteins expressed specifically in the sexual stages are important components of the oocyst wall^92^. In our study we identified eleven genes involved in protein glycosylation. The tyrosine rich proteins undergo proteolysis into smaller tyrosine-rich peptides before oocyst wall assembly^88^. In the present study, 36 genes coding for enzymes proposed to be involved in the proteolysis of dityrosine bond formation in the oocyst wall were identified, including, among others, five subtilisins, four peptidases, seven proteasome units, four microneme and two pan domain-proteins. The subtilisins are particularly interesting with regard to dityrosine bond formation^8^. Cross-linking of the smaller tyrosine-rich proteins to dityrosine imparts further stability to the oocyst wall, and the role of peroxidases in catalysing this reaction is implicated^93^. A total of 37 proteins involved in oxidoreductase activities were identified. The oocyst wall consists mainly of proteins and lipids^39^. Polysaccharide granules and lipid droplets are also found in the cytoplasm of mature macrogamonts in *T. gondii* and *E. maxima*^39,94,95^. Recent studies indicate that the coccidian oocyst wall architecture is comprised not only of glycoproteins but also of an outer layer of acid-fast lipids^39^. Nineteen genes coding for proteins with predicted roles in synthesis, metabolim or remodeling of acid-fast lipids were found in *C. suis*. Consistent with the presence of triglycerides in oocyst walls, mRNAs of diacylglycerol acyltransferases and three putative acyl coenzyme A (acyl-CoA) cholesterol acyltransferases were also found to be upregulated. The roles of these putative proteins in sexual stage biology need to be further investigated. Additional proteins considered to be of interest were surface proteins and proteins involved in oocyst wall resistance. Among these are nine surface proteins, including three SAGs, two fasciclins and three proteophosphoglycans, one outer omp85 family protein, one longevity-assurance domain-containing protein and three late embryogenesis abundant domain proteins (LEA). All these proteins were previously identifed in *T. gondii* oocysts and in *Eimeria* gametocytes and oocysts^14,20,38,89^ (Tables 1 and S5).

**Table 1:** Upregulated transcripts coding for proteins with known or putative roles in oocyst wall composition and surface.

Gene ID	logFC	FDR_adj_pval	Annotation	Comparison	Function
CSUI_008806	4.62	1,47E+04	oocyst wall protein	UT12_UT23_UT13	Oocyst wall
CSUI_006207	1.75	1,04E+08	oocyst wall protein	UT23_UT13	Oocyst Wall
CSUI_002027	5.47	1,88E+04	toxoplasma gondii family a protein	UT12_UT23_UT13	Oocyst Wall
CSUI_006655	1.78	9,10E+06	toxoplasma gondii family a protein	UT12_UT13	Oocyst Wall
CSUI_010157	4.32	4,37E+05	toxoplasma gondii family a protein	UT12_UT23_UT13	Oocyst Wall
CSUI_003908	3.39	2,68E+06	toxoplasma gondii family d protein	UT12_UT13	Oocyst Wall
CSUI_004489	3.29	2,89E+05	toxoplasma gondii family d protein	UT12_UT13	Oocyst Wall
CSUI_004212	2.21	1,92E+07	toxoplasma gondii family d protein	UT23_UT13	Oocyst Wall
CSUI_009196	1.67	2,45E+09	toxoplasma gondii family d protein	UT13	Oocyst Wall
CSUI_000190	5.84	3,56E+03	hypothetical protein-TyRP	UT12_UT23_UT13	Oocyst Wall
CSUI_001473	2.77	9,93E+05	hypothetical protein-TyRP	UT12_UT13	Oocyst Wall
CSUI_001475	3.46	1,07E+05	hypothetical protein-TyRP	UT12_UT13	Oocyst Wall
CSUI_007070	4.06	2,69E+03	fasciclin domain protein	UT12_UT23_UT13	Surface
CSUI_006459	4.41	3,07E+04	fasciclin domain-containing protein	UT12_UT23_UT13	Surface
CSUI_002476	1.08	4,52E+09	outer omp85 family protein	UT13	Surface
CSUI_006179	3.96	3,97E+01	SAG domain-containing protein	UT12_UT23_UT13	Surface
CSUI_004248	3.06	1,29E+06	sag-related sequence srs26i	UT12_UT23_UT13	Surface
CSUI_005667	1.57	3,74E+08	sag-related sequence srs28	UT23_UT13	Surface
CSUI_009850	3.09	6,33E+08	proteophosphoglycan related	UpT12_UpT13	Surface
CSUI_006635	1.68	7,28E+05	proteophosphoglycan related	UpT23_UpT13	Surface
CSUI_001278	2.85	3,81E+08	proteophosphoglycan related protein	UpT12_UpT13	Surface
CSUI_001693	1.11	1,10E+08	longevity-assurance protein domain-containing protein	UT23	Resistance
CSUI_001900	5.63	2,06E+03	late embryogenesis abundant domain protein	UT12_UT23_UT13	Resistance
CSUI_001899	5.31	1,95E+04	late embryogenesis abundant domain protein	UT12_UT23_UT13	Resistance
CSUI_005059	1.46	4,91E+09	late embryogenesis abundant domain protein	UT13	Resistance

They are listed along with their transcript abundance (LogFC), annotation, comparison (upregulated transcripts (UT) in early sexual stages(2) compared to merozoites(1), UT12, late sexual stages (3) compared to merozoites (1), UT13, and late sexual stages (3) compared to early sexual stages(2), UT23) and biological function.

Most of the genes identified are related to macrogamete development and oocyst formation, predominating during the transition from asexual to sexual stages in cell culture. GAM are well characterized tyrosine-rich proteins of the oocyst wall of *Eimeria*^82,95,96^; they were previously developed as antigens for transmission-blocking vaccines targeting the gametocyte-specific proteins GAM56, GAM82 and GAM22^95,97–100^. These proteins are potent immunogens for the use as vaccines against chicken coccidiosis as they induce a diverse and robust immunity^101^. In *Plasmodium*, vaccines incorporating magrogamete surface antigens significantly reduced oocyst formation, and several surface proteins of *Plasmodium* macrogametes such as Pfs25 and Pfs230 are investigated in ongoing trials^102–104^. All these findings indicate that inhibiting the fertilization of macrogametes by microgametes and the oocyst wall formation can effectively interfere with the parasite’s developmental cycle.

### Microgamete-specific genes

Scanning electron microscopy observations of *C. suis* showed that microgametes consisted of a small, spherical body with two opposing flagella^7^. The molecular characterisation of microgametes in the Coccidia is still limited. Microgametes use flagella to move quickly in search of macrogametes and fertilize them, leading to the formation of the zygote. Although there are few data on the molecular mechanisms underlying this development, some proteins and genes were predicted to be involved in DNA replication, microgamete budding from microgamonts, axoneme/flagellar formation and gamete fusion^14,17,20,105,106^. We detected 46 upregulated transcripts coding for proteins with a putative role in microgamete biology. Of these, CSUI_004019 and CSUI_006055 were the two most abundant transcripts, coding for a tubulin beta-chain and a flagella-associated protein. In sexual stages, tubulins are the building block proteins of the microtubules that form the flagellar axoneme, basal bodies, and centrioles. They are components of the flagella that are essential for microgamete motility and fertilisation. We also found six proteins involved in motor activity and microtubule movement, 25 axonema and microtubule-associated proteins, four involved in cell budding and one in gamete fusion (Table 2 and S6).

**Table 2:** Upregulated transcripts coding for proteins with known or putative roles in microgamete biology.

Gene ID	logFC	FDR_adj_pval	Annotation	Comparison	Function
CSUI_007002	1.23	5,46E+09	centrin 2	UT23_UT13	Flagella
CSUI_006055	4.27	3,82E+03	flagellar associated protein	UT12_UT23_UT13	Flagella
CSUI_006910	2.80	3,12E+05	flagellar associated protein	UT12_UT23_UT13	Flagella
CSUI_003885	1.23	8,85E+08	kinesin motor domain-protein	UT23_UT13	Microtubule
CSUI_005407	1.39	4,18E+09	myosin a	UT13	Microtubule
CSUI_009311	1.96	2,06E+08	myosin heavy chain	UT23_UT13	Microtubule
CSUI_000425	1.38	5,37E+03	myosin k	UT13	Microtubule
CSUI_009385	1.18	2,96E+03	myosin k	UT13	Microtubule
CSUI_005546	1.10	2,12E+09	myosin light chain	UT13	Microtubule
CSUI_011403	1.20	5,16E+0	myosin regulatory light chain	UT13	Microtubule
CSUI_000854	1.58	1,43E+07	non-muscle myosin heavy	UT23_UT13	Microtubule
CSUI_007953	3.02	1,69E+05	chromosome-associated kinesin klp1	UT12_UT23_UT13	Microtubule
CSUI_007586	3.41	8,12E+06	dynein gamma flagellar outer	UT12_UT23_UT13	Axonema
CSUI_004717	1.62	7,86E+07	dynein light chain dlc	UT23_UT13	Axonema
CSUI_001333	1.08	1,22E+04	dynein light chain roadblock-type 2	UT13	Axonema
CSUI_002604	2.73	7,88E+05	growth arrest-specific protein 8	UT12_UT23_UT13	Axonema
CSUI_000245	2.21	4,06E+07	heavy chain 2 family protein	UT23_UT13	Axonema
CSUI_000472	3.19	2,40E+08	male gamete fusion factor	UT12_UT23_UT13	Gamete fusion
CSUI_002998	3.20	1,95E+04	morn repeat-containing protein	UT12_UT23_UT13	Cell budding
CSUI_000048	1.30	2,22E+07	morn repeat-containing protein	UT13	Cell budding
CSUI_004816	1.70	2,78E+06	morn repeat-containing protein	UT23_UT13	Cell budding

They are listed along with their transcript abundance (LogFC), annotation, comparison (upregulated transcripts (UT) in early sexual stages(2) compared to merozoites(1), UT12, late sexual stages (3) compared to merozoites (1), UT13, and late sexual stages (3) compared to early sexual stages(2), UT23) and biological function.

Based on the motile nature of the male sexual stages and the lack of invasion machinery genes in sexual stages, it is obvious that the fertilization process takes place extracellularly, rather than intracellularly as previously assumed^40,107,108^. The transmission blocking potential of proteins specific to sexual stage as candidates for vaccination or drug targets has been suggested in related Coccidia and other Apicomplexa^2^. Oral application of sera containing *E. tenella* gamont-specific monoclonal antibodies significantly reduced oocyst output and cecal lesions in chicken^109^. Studies in *Plasmodium* proposed the HAP2 fusion protein as a candidate for a transmission-blocking vaccine^110–112^. Recently, a HAP2-deficient *T. gondii* strain was created using the CRISPR/Cas9 approach and used as transmission blocking control strategy by immunising cats against a challenge with a *T. gondii* wildtype strain^22^. This in turn supports the assumption that intestinal sexual stages are accesible for specific antibodies which could be induced by vaccination or transferred by colostrum (as maternal antibodies). For *C. suis* it was previously shown that high levels of colostral and possibly milk antibodies from superinfected sows exert significant protection of suckling piglets against experimental *C. suis* infection^113^. Although these antibodies were not characterised regarding the targeted proteins or parasite stages, it is conceivable that sexual-stage specific proteins could be implemented a vaccine targets in this context.

### Inmunolocalization of CSUI_001473 antigens in macrogametes and oocysts

The oocyst wall is a distinctive characteristic of coccidian development and the key stage of transmission^39^. It is described that vaccines incorporating antigens from magrogamete surface or oocyst wall significantly reduced the oocyst formation. We hypothesized that targeting these stages may be an effective approach in *C. suis* parasite control in the future. In our previous study applying qRT-PCR on stages derived from in vitro cultures of *C. suis*, transcrip levels of CSUI_001473 (*Cs*TyRP) were highly upregulated with a peak on day 13 of in vitro culture or on day 4 of transfer to host-cell free medium and declined after that^7,11^, correlating with the distinct upregulation of transcript level in the current analysis. We selected CSUI_001473 to test the proof of principle that targeting a sexual stage specific antigen could be used as a candidate for a transmission-blocking vaccine.

A single 1463 bp CSUI_001473 open reading frame encoded a protein of 353 amino acids with the predicted molecular mass of 39 kDa. The deduced amino acid sequence had a predicted N-terminal 19-amino acids signal peptide for entrance into the secretory pathway. No predicted transmembrane domains were identifed. The recombinant CSUI_001473 protein (rCSUI_001473) revealed a major protein band of ~ 55 kDa, higher than the predicted 46 kDa (Figure S1a), after induction with 1 mM IPTG for 4 h at 37 °C. Purification was performed under denaturing conditions. These antibodies recognized a single strong band of approximately 55 kDa, corresponding to rCSUI_001473, and a lower molecular weight protein band which might be degraded products or truncations of rCSUI_001473 (Figure S1b). Furthermore, to confirm that the chicken anti-rCSUI_001473 serum recognized the native form of CSUI_001473 protein, a crude extract of sexual stage proteins was probed with anti-rCSUI_001473 serum in which a band of aproximatively 48 kDa was recognized. As expected, negative chicken serum failed to detect any bands of the expected size in Western blot (Figure S1c and d).

**Figure 5:**
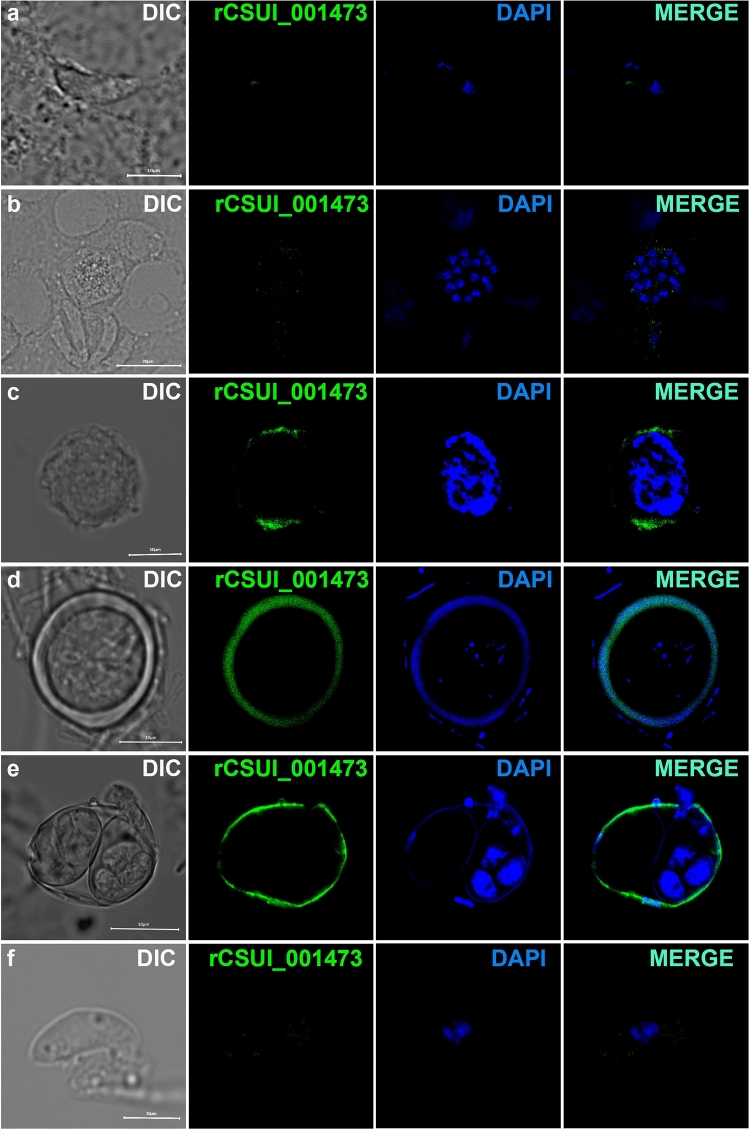
Localization of CSUI_001473 antigens in differents *C. suis* stages. (**a**) Merozoite from day 6 of in vitro culture. (**b**) Microgamont from day 9 of culture. (**c**) Macrogamont from day 9 of culture. (**d**) Unsporulated oocyst from day 14. (**e**) Sporulated oocyst ex vivo (isolated from the feces of experimentally infected piglets). (**f**) Sporozoite released from in vitro excysted oocysts. DIC, differential interference contrast microscopy; DAPI staining appears in blue; green indicates binding of anti-rCSUI-001473 antibodies, and turquoise indicates merged results. Scale bar = 10 μm.

To test the hypothesis that CSUI_001473 is a component of the oocyst wall we performed immunolocalisation studies, again using chicken anti-rCSUI_001473 serum. The protein localized to *C. suis* macrogametes (Fig. 5), specifically to the periphery of the parasite cell, and to the outer wall of the unsporulated and sporulated oocyst, but not to the the sporocyst wall. We did not detect antibody binding in merozoites or microgamonts. This confirms that CSUI_001473 is homologous to the proteins identified in the oocyst proteome of *T. gondii* and is an oocyst wall protein member.

### Serum inhibition assay

**Figure 6:**
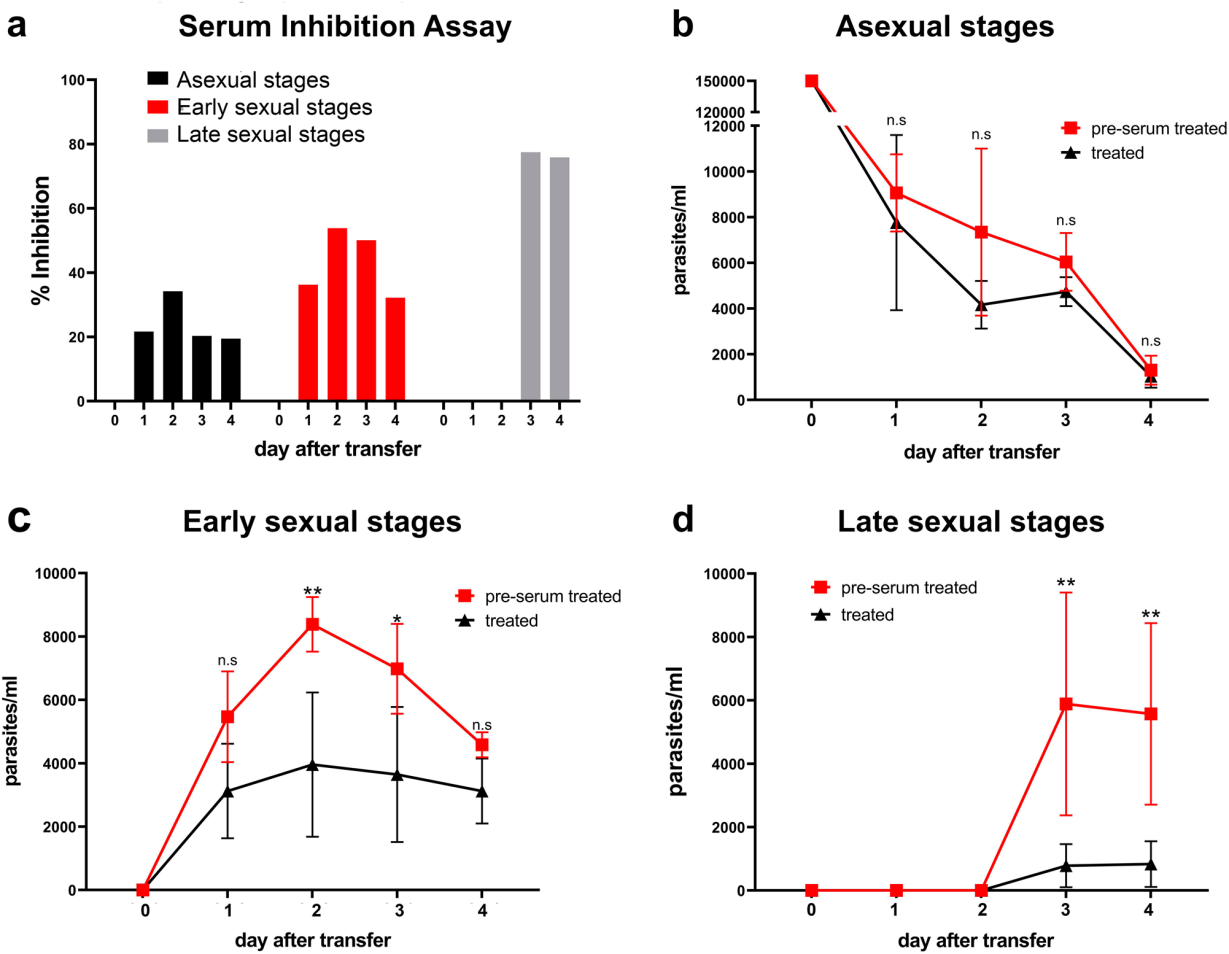
Serum Inhibition assay. (**a**) Inhibition rates for asexual, early (mostly gamonts) and late (mostly gametes) sexual stages of *C. suis* in a host-cell free culture 0–4 days after transfer of merozoites. (**b** to **d**) Total numbers of counted stages by day of cultivation in host-cell free culture. n.s.: not singificant, *: *P* ≤ 0.05, **: *P* ≤ 0.01. Values represent the mean ± standard deviation (SD) from three independent experiments. n.s.: not singificant, *: *P* ≤ 0.05, **: *P* ≤ 0.01.

No genetic manipulation technique is currently available for *C. suis* to confirm the direct involvement of CSUI_001473 in oocyst formation and/or development. In order to test whether CSUI_001473 expression is essential for oocyst wall formation we tested whether chicken anti-rCSUI_001473 serum can inhibit late sexual stage development. The novel host cell-free in vitro culture system for *C. suis* made it possible to evaluate the effects of culture conditions on the development of merozoites to sexual stages and oocysts^11^. The addition of antiserum did not significantly decrease the number of asexual stages compared to the pre-immune serum, merozoite growth was inhibited by only 20% (Fig. 6a and b). The numbers of newly developed early sexual stages increased until three days after merozoite transfer, the late sexual stages (macrogametes and free motile microgametes) could be detected from three days after transfer, and both unsporulated and sporulated oocysts were present by day four post transfer. Treatment with positive serum significantly inhibited the development of early and late sexual stages (Fig. 6c and d). Development of early sexual stages was inhibited by 50%, while the late sexual stages were reduced by 75% (Fig. 6a).

Taken together our previous studies with the current results on the protein localization on the surface of macrogametes and oocyst wall and the development inhibition of early and late sexual stages confirm that CSUI_001473 transcripts encode a protein that plays a decisive functional role in the development and/or formation of the oocyst wall.

## Conclusions

A comparative RNAseq transcriptomics approach led to the identification of genes specifically expressed in *C. suis* early and late sexual stages (gamonts and gametes) in comparison to asexual stages (merozoites) in vitro. We could describe global changes in gene expression during sexual differentiation and gamete maturation from merozoites to gametes and oocysts in vitro. This set of results represents a detailed overview of the biology of sexual development in this model coccidian in comparison to asexual intracellular replication. In addition, a previously uncharacterized protein of the oocyst wall of *C. suis* was investigated that may represent a candidate for a transmission-blocking vaccine against piglet cystoisosporosis. These new findings create a dataset that incorporates an initial comprehensive view of the mechanisms associated with sexual reproduction and oocyst formation in a range of taxa as a common denominator in the understanding of parasite biology and definition of intervention targets.

## Materials and methods

### *Cystoisospora suis* oocyst collection

*Cystoisospora suis* oocysts (strain Wien I) were obtained from experimentally infected suckling piglets as described previously^7,10^. Piglets were raised with the sow in the animal facilities of the Institute of Parasitology, University of Veterinary Medicine Vienna, Austria.

### In vitro culture

Intestinal porcine epithelial cells (IPEC-1, ACC 705, Leibniz Institute DSMZ-German Collection of Microorganisms and Cell Cultures GmbH, Leibniz, Germany) were used as host cells in vitro and seeded in a density of 4 × 10^5^ cells per well in a 6-well plate (PAA, Pasching, Austria). A total of 21 plates were used with material from three pooled plates constituting a biological replicate). Cells were grown in in DMEM/Ham’s F-12 medium (Gibco—Fisher Scientific GmbH, Schwerte, Germany) with 5% fetal calf serum (Gibco) and 100 U/ml penicillin and 0.1 mg/ml streptomycin (PAA, Pasching, Austria) at 37 °C in 5% CO_2_. After 24 h of cell growth IPEC-1 cells were infected with 5 × 10^3^ sporozoites/well released from excysted oocysts and incubated further at 40 °C under 5% CO_2_^5,10^.

### Experimental design, sampling and RNA-seq library preparation

For the sampling of sexual stages released from host cells we collected cell culture supernatant every day, from day of cultivation (doc) 6 to day 14. The material was washed twice with phosphate-buffered saline (PBS; Gibco) and pelleted by centrifugation at 600 × g for 10 min. The numbers of merozoites, sexual stages and oocysts were counted in a Neubauer-counting chamber for each given time point. For each day, seven biological replicates were harvested and the mean numbers of each stage per biological replicate were calculated.

Pellets from the same wells were pooled to increase the number of parasites per sample and the analysis was performed for three time points:

1. pool of days 6, 7 and 8 (merozoites, type I and II) = Time point 1.
2. pool of days 9, 10 and 11 (merozoites type II and early sexual stages, i.e. gamonts) = Time point 2.
3. pool of days 12, 13 and 14 (mainly sexual stages, gametes, and unsporulated oocysts) = Time point 3.

Total RNA was isolated from infected cell cultures using an RNeasy® Mini kit (Qiagen, Hilden, Germany) and treated with RNase-free DNase (Qiagen) according to the manufacturer’s instructions to remove any DNA contamination. Total RNA was quantified using a NanoDrop® 2000 (Thermo Fischer Scientific, Waltham, MA, USA), and samples were sent for library preparation using a reverse stranded protocol with poly-A enrichment. Sequencing libraries were prepared at the Core Facility Genomics, Medical University of Vienna, using the NEBNext Poly(A) mRNA Magnetic Isolation Module® and the NEBNext Ultra® II Directional RNA Library Prep Kit for Illumina according to manufacturer's protocols (New England Biolabs, Ipswich, Massachusetts, USA). Libraries were QC-checked on a Bioanalyzer 2100® (Agilent Technologies, Santa Clara, CA, USA) using a High Sensitivity® DNA kit for correct insert size and quantified using Qubit dsDNA HS® assay (Invitrogen, Waltham, Massachusetts, USA). Pooled libraries were sequenced on a NextSeq500® instrument (Illumina, San Diego, California, USA) in 1 × 75 bp single-end sequencing mode. Approximately 21.5 million reads were generated per sample.

### RNA-Seq data analysis

Sequencing reads were mapped against the concatenated fasta sequences of *C. suis* (version 48 from ToxoDB)^114^ and *S. scrofa* (version 1.11 from Ensembl, GCA_000003025.6)^115,116^ using STAR (version 2.7.3a with option –outSAMmultNmax 1)^117^ and the combined annotations of each genome (version 48 for *C. suis* and 11.1.98 for *S. scrofa*). Only the reads mapping to the *C. suis* genome were subsequently used for quantification and further analysis.

Quality control was performed with FastQC^118^ and QualiMap^119^. RNA degradation was taken into account via the TIN (Transcript Integrity Number) values, which were measured for each gene and library with the RSeQC^120^. It was used to assess gene body coverage (module geneBody_coverage.py with with option -l 500) and to calculate transcript integrity numbers (TIN scores, module tin.py with option -c 20), TIN is considered an accurate and reliable measurement of RNA integrity at the sample level^120,121^. Gene expression was quantified with featureCounts (version 1.5.0a)^122^ with options -s 2 -Q 20 –primary.

### Identification and analysis of differentially expresed genes

All statistical analysis were performed in R (version 4.1)^123^.

Given the repeated measures design of our experiment (briefly, gene expression was measured for seven samples at each of three timepoints) we employed a linear mixed model framework^124^ to account for the covariance structure in the data. Differential gene expression analysis between the three time points was performed via linear mixed models with the function *dream* (R package variancePartition, version 1.18.3)^124,125^, which is a wrapper for the function *lmer* in package lme4. Replicate ID was fitted as random intercept, and hypothesis testing was carried out for a fixed categorical effect of time with the three time points as factor levels. We further included the median TIN scores, calculated across all genes in each library, as a continuously distributed (nuisance) covariate in our model. We filtered for genes with a minimum count of 30 and four counts per million reads in at least four out of seven replicates of each time point. The remaining counts were quantile normalized before differential gene expression analysis with the function *voomWithDreamWeights* (R package variancePartition). The *p*-values were adjusted for multiple testing according to Benjamini and Hochberg's false discovery rate (FDR) correction^126^. Genes with FDR > 0.05 and absolute log2FC > 1 were considered significantly differentially expressed.

### Gene ontology enrichment analysis

To explore the broader biological context of the identified genes, gene ontology (GO) enrichment analysis was performed via topGO^127^ with the Fisher’s Exact Test and the GO annotations from ToxoDB (version 50). The “Weighted01” algorithm which accounts for the GO hierarchy was applied.

### qRT-PCR validation of DEGs

The cDNA samples were synthesised from DNase-treated total RNA used in RNASeq. Synthesis of cDNA was accomplished using the iScript® cDNA synthesis kit (Bio-Rad, Hercules, California, USA). Quantitative PCR amplification of cDNA was carried out on a Mx3000P thermal cycler (Agilent Technologies, Santa Clara, CA, USA). The primers for gene amplification are listed in Table S7. Reaction mixtures contained 2.5 μl of sample cDNA (50 ng/μl), 5 μl of SsoAdvanced™ Universal Probes Supermix (Bio-Rad, Hercules, California, USA) and 1.3 μl of nuclease-free water with primers and probes at a final concentration of 500 and 200 nM, respectively. Activation of polymerase was performed at 95 °C for 2 min, followed by 50 cycles of 95 °C for 15 s and 60 °C for 30 s. Each sample was run in triplicate. The qPCR results were normalized against the mean of two reference genes, GAPDH and actin (see primers efficiencies in Supplemental file S1) . Average gene expression relative to the endogenous control for each sample was calculated using the 2 − ΔΔCq method. The relative fold change of gene expression was expressed as the mean and standard deviation. Statistical analysis were performed using the ANOVA one way test with the software GraphPad® Prism 9.2 (GraphPad Software, San Diego, CA). Differences were considered statistically significant at P ≤ 0.05.

### Recombinant protein expression

A Champion pET151 Directional TOPO® Expression Kit was used for the expression of recombinant proteins with N-terminal V5-6xHis tags. Coding sequences of the hypothetical gene CsTyRP (CSUI_001473) were amplified by PCR from cDNA using a Q5 high Fidelity® DNA Polymerase (New England Biolabs, Ipswich, Massachusetts, USA) according to manufacturer’s instructions. The gene-specific primers used for amplification and subsequent cloning into Champion pET151 Directional TOPO® are listed in Table S7 (primers no. 25–26). After verification of the correct cloning in BL21 Star® (DE3) and confirmation of the reading frames, plasmids with the correct inserts were used to transform One Shot® chemically competent *E. coli* (Thermo Fischer). Briefly, bacteria containing the recombinant plasmid were grown overnight in non-inducing LB medium at 37 °C on a culture shaker at 180 rpm. One milliliter of pre-cultured LB medium was then inoculated in 50 ml of fresh LB medium and incubated for 1 h at 37 °C, 220 rpm, until OD_600_ = 0.6, and the expression of the recombinant proteins was induced by adding 1 mM of IPTG (Sigma-Aldrich, St. Louis, Missouri, USA), followed by incubation for 4 h. The culture was then centrifuged at 4,000 × g for 30 min. The pellet was re-suspended in lysis buffer (20 mM Na_2_HPO_4_; 8 M urea; 0.5 M NaCl, 5 mM imidazole, pH 8) under constant stirring for 1 h for solubilization and then centrifuged at 10,000 × g for 20 min. The lysates were analysed by SDS-PAGE (12.5%) followed by staining with Coomassie blue (BioRad, Hercules, California, USA). Recombinant proteins were purified using a Ni-sepharose column (His GraviTrap®, GE Healthcare, Chicago, Illinois USA) following the manufacturer’s instructions.

### Antibody production

The recombinant protein was used for immunizing two chicken according to a standard 87-day programme immunization procedure (Eurogentec, Seraing, Liège, Belgium). Before injection (day 0), preimmune egg yolk was collected (pre-immune serum), and subsequent imunizations (100 μg of antigen per injection) were made on days 14, 28, 56 and (as an additional booster) on day 99. Egg yolks were collected during three time periods (days 38–52, days 66–81 and days 109–121). The collected egg yolk sera were evaluated in conventional ELISA for checking their respective titers. The isolation of IgY from the egg yolk and their subsequent affinity purification were performed by Eurogentec.

### Western blotting

To test the quality and specificity of the sera produced, we loaded 2 and 10 μg of the recombinant protein and total protein from cell culture samples, respectively, mixed with 2 × Laemmli sample buffer, on two 12.5% SDS-PAGE gels, one was stained them with Coomassie blue after electrophoresis, the other one was used to transferprotein bands onto a PVDF membrane (Mini ProBlott Membranes, Applied Biosystems, Foster City, CA, USA) using a Transblot device (Bio-Rad). Membrane strips was subsequently blocked for 30 min at room temperature in a TBS solution containing 1% casein and 0.05% Tween 20. After blocking, the membranes were incubated with chicken anti-rCSUI_001473 polyclonal sera, or negative chicken sera dilutions 1:500 in TTBS buffer (100 mM Tris, 0.9% NaCl, 0.1% Tween 20) at room temperature for 1 h. After rinsing with TTBS for 30 min, blots were exposed to biotinylated goat anti-chicken IgY (Vector Laboratories, Burlingame, CA, USA) as secondary antibody at 1:5000 dilution in TTBS buffer for 1 h at room temperature, incubated with avidin–biotin complex solution (Vector Laboratories) and finally detected by addition of 3,3′-5,5′-tetramethylbenzidine according to the manufacturer’s instructions (Vector Laboratories).

### Inmunofluorescence microscopy

Merozoites and gamonts from cell culture supernatants were washed once with PBS at room temperature and transferred to poly-L-lysine treated glass slides (Polysciences Inc., Hirschberg an der Bergstrasse, Germany) and air dried before fixation. Parasites were either fixed with 4% paraformaldehyde in PBS for 10 min followed by permeabilization with 0.25% TritonX-100 in PBS for 10 min or fixed in ice-cold 100% methanol for 10 min and then blocked with 4% bovine serum albumin (Sigma-Aldrich, St. Louis, Missouri, USA) in PBS for 2 h at room temperature. A 1:500 dilution of anti-rCSUI_001473 polyclonal sera was added and incubated for 2 h at room temperature followed by 1 h incubation with a 1:300 dilution of Alexa Fluor® (A488) goat anti-chicken IgY (Invitrogen, Eugene, OR, USA). The slides were washed five times with PBS for 25 min after each step described above. 4′,6-diamidino-2-phenylindole, DAPI (5 μg/ml) was included in the Fluoromount-G® mounting medium (Thermo Fischer Scientific) for nuclear staining. Imaging was carried out with a Zeiss LSM 510 Meta-confocal laser scanning microscope (× 63 oil immersion objective). Images were analyzed with Light Editions of Zen 2012 and 2009 (Carl Zeiss Microimaging GmbH, Jena, Germany).

### Inhibition of macrogametes and oocyst development by specific antibodies

To determine inhibition of sexual stage development and oocyst formation by specific antibodies in vitro, we adapted a previously developed host cell-free culture^10^ for treatment of merozoites with egg yolk-derived chicken antibodies. Free merozoites were obtained from monolayer culture supernatant of intestinal porcine epithelial cells 6 days after infection with sporozoites. Purified merozoites were counted and treated with 2 µg/ml of chicken anti-rCSUI_001473 polyclonal sera or 2 µg/ml pre-immune chicken serum as a negative control. The treated merozoites were transferred to fresh Advanced DMEM/F-12 culture medium (Gibco) supplemented with 5% fetal calf serum (Gibco) and penicillin/streptomycin plus l-glutamine (Gibco) onto a new uncoated ibidi 8-well ibiTreat® μ-slide (ibidi, Gräfelfing, Germany) at a concentration of 1.2 × 10^5^ merozoites per mL medium and were incubated at 40 °C under 5% CO_2_. The development of parasite stages was monitored daily. The numbers of asexual and early and late sexual stages and oocysts were monitored from the first day post transfer onwards. The numbers of stages were estimated in the host cell-free culture chambers and 10 μL of each well was counted in a Neubauer chamber at each given time point for calculation of the average numbers of sexual stages. Statistical analysis were performed using a multiple unpaired t-test with the software GraphPad® Prism 9.2 (GraphPad Software). Differences were considered statistically significant at *P* ≤ 0.05 (*).

To show significance between the average number of stages on different culture days a multiple t-test was performed. In vitro inhibition percentage for each stage was calculated as follows:

$$\% \, \mathrm{inhibition}=100\times \left(1-\frac{\mathrm{average}\, \mathrm{no}.\, \mathrm{ of \, \,parasite\,\, stages\,\, in \,\,treated \,\, cultures}}{\mathrm{average\,\, no}.\mathrm{\, of \,\,parasite \,\,stages\,\, in \,\,untreated \,\,control \,\,cultures}}\right)$$

### Gene annotation analyses

Gene annotations available on www.toxodb.org were used for *C. suis* genes described in this study. The identification of potential homologues of *C. suis* hypothetical proteins was also carried out using a BLAST analyses on www.toxodb.org and www.plasmodb.org.

### Ethics approval

All procedures in this study involving experimental animals were approved by the institutional ethics and animal welfare committeee and the national authority according to §§26ff. of the Animal Expriments Act, Tierversuchsgesetz 2021—TVG 2012 und der number 2021–0.030.760.. All efforts were made to minimize the number of animals used for *C. suis* oocyst generation. All methods were performed in accordance with the guidelines and regulations approved by University of Veterinary Medicine Vienna and the national authority (Austrian Federal Ministry of Science, Health and Economy). The study is reported in accordance with ARRIVE guidelines.

## Supplementary Information

Supplementary Information 1.

Supplementary Information 2.

Supplementary Information 3.

Supplementary Information 4.

Supplementary Information 5.

Supplementary Information 6.

Supplementary Information 7.

Supplementary Information 8.

Supplementary Information 9.

Supplementary Information 10.

Supplementary Information 11.

## Data Availability

All data are contained in the publication.

## References

[1] Barta J, Schrenzel M, Carreno R, Rideout B (2005). The Genus *Atoxoplasma* (Garnham 1950) as a Junior Objective Synonym of the Genus *Isospora* (Schneider 1881) Species Infecting Birds and Resurrection of *Cystoisospora* (Frenkel 1977) as the Correct Genus for *Isospora* Species Infecting Mammals. J. Parasitol..

[2] Cruz-Bustos T, Feix AS, Ruttkowski B, Joachim A (2021). Sexual development in non-human parasitic apicomplexa: just biology or targets for control?. Anim. Open Access J. MDPI.

[3] Lindsay DS, Dubey JP, Blagburn BL (1997). Biology of *Isospora* spp. from humans, nonhuman primates, and domestic animals. Clin. Microbiol. Rev..

[4] Joachim A, Shrestha A, Dubey JP (2019). Coccidiosis of pigs. Coccidiosis in livestock, poultry, companion animals, and humans.

[5] Shrestha A (2015). *Cystoisospora suis—*A model of mammalian cystoisosporosis. Front. Vet. Sci..

[6] Shrestha A (2020). Shifts in the fecal microbial community of *Cystoisospora suis* infected piglets in response to Toltrazuril. Front. Microbiol..

[7] Feix AS, Cruz-Bustos T, Ruttkowski B, Joachim A (2020). Characterization of *Cystoisospora suis* sexual stages in vitro. Parasit. Vectors.

[8] Mai K (2009). Oocyst wall formation and composition in coccidian parasites. Mem. Inst. Oswaldo Cruz..

[9] Weedall GD, Hall N (2015). Sexual reproduction and genetic exchange in parasitic protists. Parasitology.

[10] Worliczek HL (2013). *Isospora suis* in an epithelial cell culture system - an in vitro model for sexual development in coccidia. PLoS ONE.

[11] Feix AS (2021). Progression of asexual to sexual stages of *Cystoisospora suis* in a host cell-free environment as a model for Coccidia. Parasitology.

[12] Novaes J (2012). A comparative transcriptome analysis reveals expression profiles conserved across three *Eimeria* spp. of domestic fowl and associated with multiple developmental stages. Int. J. Parasitol..

[13] Wang X (2019). RNA Sequencing analysis of chicken cecum tissues following *Eimeria tenella* infection in vivo. Genes (Basel).

[14] Walker RA (2015). RNA Seq analysis of the *Eimeria tenella* gametocyte transcriptome reveals clues about the molecular basis for sexual reproduction and oocyst biogenesis. BMC Genomics.

[15] Amiruddin N (2012). Characterisation of full-length cDNA sequences provides insights into the *Eimeria tenella* transcriptome. BMC Genomics.

[16] Su S (2017). Comparative transcriptome analysis of second- and third-generation merozoites of *Eimeria necatrix*. Parasit. Vectors.

[17] Su S (2018). Comparative transcriptome analysis of *Eimeria necatrix* third-generation merozoites and gametocytes reveals genes involved in sexual differentiation and gametocyte development. Vet. Parasitol..

[18] Behnke MS (2010). Coordinated progression through two subtranscriptomes underlies the Tachyzoite cycle of *Toxoplasma gondii*. PLoS ONE.

[19] Radke JR (2005). The transcriptome of *Toxoplasma gondii*. BMC Biol..

[20] Fritz HM (2012). Transcriptomic analysis of *Toxoplasma* development reveals many novel functions and structures specific to sporozoites and oocysts. PLoS ONE.

[21] Chen L-F (2018). Comparative studies of *Toxoplasma gondii* transcriptomes: insights into stage conversion based on gene expression profiling and alternative splicing. Parasit. Vectors.

[22] Ramakrishnan C (2019). An experimental genetically attenuated live vaccine to prevent transmission of *Toxoplasma gondii* by cats. Sci. Rep..

[23] Xue Y (2020). A single-parasite transcriptional atlas of *Toxoplasma Gondii* reveals novel control of antigen expression. Elife.

[24] Garfoot AL, Wilson GM, Coon JJ, Knoll LJ (2019). Proteomic and transcriptomic analyses of early and late-chronic *Toxoplasma gondii* infection shows novel and stage specific transcripts. BMC Genomics.

[25] Horcajo P (2018). Integrative transcriptome and proteome analyses define marked differences between *Neospora caninum* isolates throughout the tachyzoite lytic cycle. J. Proteomics.

[26] Jiménez-Meléndez, A., Ramakrishnan, C., Hehl, A. B., Russo, G. & Álvarez-García, G. RNA-Seq Analyses Reveal That Endothelial Activation and Fibrosis Are Induced Early and Progressively by *Besnoitia besnoiti* Host Cell Invasion and Proliferation . *Frontiers in Cellular and Infection Microbiology* **10**, (2020). 10.3389/fcimb.2020.00218 PMC7242738 32500038

[27] Matos LVS, McEvoy J, Tzipori S, Bresciani KDS, Widmer G (2019). The transcriptome of *Cryptosporidium* oocysts and intracellular stages. Sci. Rep..

[28] Le Roch KG (2003). Discovery of gene function by expression profiling of the malaria parasite life cycle. Science (80-).

[29] Chappell L (2020). Refining the transcriptome of the human malaria parasite *Plasmodium falciparum* using amplification-free RNA-seq. BMC Genomics.

[30] Otto TD (2010). New insights into the blood-stage transcriptome of *Plasmodium falciparum* using RNA-Seq. Mol. Microbiol..

[31] Prajapati SK (2020). The transcriptome of circulating sexually committed *Plasmodium falciparum* ring stage parasites forecasts malaria transmission potential. Nat. Commun..

[32] Silvestrini F (2005). Genome-wide identification of genes upregulated at the onset of gametocytogenesis in *Plasmodium falciparum*. Mol. Biochem. Parasitol..

[33] van Biljon R (2019). Hierarchical transcriptional control regulates *Plasmodium falciparum* sexual differentiation. BMC Genomics.

[34] Ogedengbe, M. *et al.* Molecular phylogenetic analyses of tissue coccidia (sarcocystidae; apicomplexa) based on nuclear 18s RDNA and mitochondrial COI sequences confirms the paraphyly of the genus Hammondia. *Parasitol. Open* **2**, (2016).

[35] Samarasinghe B, Johnson J, Ryan U (2008). Phylogenetic analysis of *Cystoisospora* species at the rRNA ITS1 locus and development of a PCR-RFLP assay. Exp. Parasitol..

[36] Schneider P, Reece SE (2021). The private life of malaria parasites: Strategies for sexual reproduction. Mol. Biochem. Parasitol..

[37] Smith RC, Vega-Rodríguez J, Jacobs-Lorena M (2014). The *Plasmodium* bottleneck: malaria parasite losses in the mosquito vector. Mem. Inst. Oswaldo Cruz..

[38] Possenti A (2013). Global proteomic analysis of the oocyst/sporozoite of *Toxoplasma gondii* reveals commitment to a host-independent lifestyle. BMC Genomics.

[39] Belli SI, Smith NC, Ferguson DJP (2006). The coccidian oocyst: a tough nut to crack!. Trends Parasitol..

[40] Walker RA, Ferguson DJP, Miller CMD, Smith NC (2013). Sex and *Eimeria*: a molecular perspective. Parasitology.

[41] Martorelli Di Genova B, Knoll LJ (2020). Comparisons of the sexual cycles for the coccidian parasites *Eimeria* and *Toxoplasma*. Front. Cell. Infect. Microbiol..

[42] Ramakrishnan C, Smith NC (2021). Recent achievements and doors opened for coccidian parasite research and development through transcriptomics of enteric sexual stages. Mol. Biochem. Parasitol..

[43] Attias M (2020). The life-cycle of *Toxoplasma gondii* reviewed using animations. Parasit. Vectors.

[44] Dubois DJ, Soldati-Favre D (2019). Biogenesis and secretion of micronemes in *Toxoplasma gondii*. Cell. Microbiol..

[45] Wang J-L (2017). Functional Characterization of Rhoptry Kinome in the Virulent *Toxoplasma gondii* RH Strain. Front. Microbiol..

[46] Dubremetz JF (2007). Rhoptries are major players in *Toxoplasma gondii* invasion and host cell interaction. Cell. Microbiol..

[47] Mercier, C., Cesbron-Delauw, M. & Ferguson, D. Dense Granules of the Infectious Stages of *Toxoplasma gondii*: Their Central Role in the Host-Parasite Relationship, In Toxoplasma. (2007).

[48] Shen B, Sibley LD (2012). The moving junction, a key portal to host cell invasion by apicomplexan parasites. Curr. Opin. Microbiol..

[49] Frénal K, Dubremetz J-F, Lebrun M, Soldati-Favre D (2017). Gliding motility powers invasion and egress in Apicomplexa. Nat. Rev. Microbiol..

[50] Sibley LD (2004). Intracellular parasite invasion strategies. Science (80-)..

[51] Håkansson S, Morisaki H, Heuser J, Sibley LD (1999). Time-lapse video microscopy of gliding motility in *Toxoplasma gondii* reveals a novel, biphasic mechanism of cell locomotion. Mol. Biol. Cell.

[52] Heintzelman MB (2015). Gliding motility in apicomplexan parasites. Semin. Cell Dev. Biol..

[53] Kato K (2018). How does *Toxoplama gondii* invade host cells?. J. Vet. Med. Sci..

[54] Bargieri DY (2013). Apical membrane antigen 1 mediates apicomplexan parasite attachment but is dispensable for host cell invasion. Nat. Commun..

[55] Mital J, Meissner M, Soldati D, Ward GE (2005). Conditional expression of *Toxoplasma gondii* apical membrane antigen-1 (TgAMA1) demonstrates that TgAMA1 plays a critical role in host cell invasion. Mol. Biol. Cell.

[56] Harding CR, Meissner M (2014). The inner membrane complex through development of *Toxoplasma gondii* and *Plasmodium*. Cell. Microbiol..

[57] Ferreira JL (2021). The dynamic roles of the inner membrane complex in the multiple stages of the malaria parasite. Front. Cell. Infect. Microbiol..

[58] Harding CR, Frischknecht F (2020). The riveting cellular structures of apicomplexan parasites. Trends Parasitol..

[59] Frénal K, Soldati-Favre D (2013). Un complexe moléculaire unique à l’origine de la motilité et de l’invasion des Apicomplexes. Med. Sci..

[60] Boucher LE, Bosch J (2015). The apicomplexan glideosome and adhesins - Structures and function. J. Struct. Biol..

[61] Tosetti N (2020). Essential function of the alveolin network in the subpellicular microtubules and conoid assembly in *Toxoplasma gondii*. Elife.

[62] Lourido S, Moreno SNJ (2015). The calcium signaling toolkit of the Apicomplexan parasites *Toxoplasma gondii* and *Plasmodium* spp. Cell Calcium.

[63] Uboldi AD, Wilde M-L, Bader SM, Tonkin CJ (2021). Environmental sensing and regulation of motility in *Toxoplasma*. Mol. Microbiol..

[64] Hehl AB (2015). Asexual expansion of *Toxoplasma gondii* merozoites is distinct from tachyzoites and entails expression of non-overlapping gene families to attach, invade, and replicate within feline enterocytes. BMC Genomics.

[65] Blader IJ, Saeij JP (2009). Communication between *Toxoplasma gondii* and its host: impact on parasite growth, development, immune evasion, and virulence. APMIS.

[66] Lekutis C, Ferguson DJP, Grigg ME, Camps M, Boothroyd JC (2001). Surface antigens of *Toxoplasma gondii*: variations on a theme. Int. J. Parasitol..

[67] Craver MPJ, Rooney PJ, Knoll LJ (2010). Isolation of *Toxoplasma gondii* development mutants identifies a potential proteophosphogylcan that enhances cyst wall formation. Mol. Biochem. Parasitol..

[68] Tomita T (2013). The *Toxoplasma gondii* Cyst wall protein CST1 is critical for cyst wall integrity and promotes bradyzoite persistence. PLOS Pathog..

[69] Sharma J, Rodriguez P, Roy P, Guiton PS (2020). Transcriptional ups and downs: patterns of gene expression in the life cycle of *Toxoplasma gondii*. Microbes Infect..

[70] Pinckney RD, Lindsay DS, Toivio-Kinnucan MA, Blagburn BL (1993). Ultrastructure of *Isospora suis* during excystation and attempts to demonstrate extraintestinal stages in mice. Vet. Parasitol..

[71] Lindsay DS, Houk AE, Mitchell SM, Dubey JP (2014). Developmental Biology of *Cystoisospora* (Apicomplexa: Sarcocystidae) Monozoic Tissue Cysts. J. Parasitol..

[72] Ferreira R, Borges-Silva W, de Jesus RF, Gondim LFP (2019). Development of *Cystoisospora felis* in cell culture and in vitro formation of monozoic tissue cysts. Front. Vet. Sci..

[73] Dubey JP (2019). Re-evaluation of merogony of a *Cystoisospora ohioensis*-like coccidian and its distinction from gametogony in the intestine of a naturally infected dog. Parasitology.

[74] Riechmann JL, Meyerowitz EM (1998). The AP2/EREBP family of plant transcription factors. Biol. Chem..

[75] Balaji S, Babu MM, Iyer LM, Aravind L (2005). Discovery of the principal specific transcription factors of Apicomplexa and their implication for the evolution of the AP2-integrase DNA binding domains. Nucleic Acids Res..

[76] Painter HJ, Campbell TL, Llinás M (2011). The Apicomplexan AP2 family: integral factors regulating *Plasmodium* development. Mol. Biochem. Parasitol..

[77] Ralph SA, Cortés A (2019). *Plasmodium* sexual differentiation: how to make a female. Mol. Microbiol..

[78] Josling GA (2020). Dissecting the role of PfAP2-G in malaria gametocytogenesis. Nat. Commun..

[79] Neveu G, Beri D, Kafsack BFC (2020). Metabolic regulation of sexual commitment in *Plasmodium falciparum*. Curr. Opin. Microbiol..

[80] Radke JB (2018). Transcriptional repression by ApiAP2 factors is central to chronic toxoplasmosis. PLoS Pathog..

[81] Sokol-Borrelli SL, Coombs RS, Boyle JP (2020). A Comparison of Stage Conversion in the Coccidian Apicomplexans *Toxoplasma gondii, Hammondia hammondi*, and *Neospora caninum*. Front. Cell. Infect. Microbiol..

[82] Wallach MG (1989). *Eimeria maxima*: Identification of gametocyte protein antigens. Exp. Parasitol..

[83] Spano F, Puri C, Ranucci L, Putignani L, Crisanti A (1997). Cloning of the entire COWP gene of *Cryptosporidium parvum* and ultrastructural localization of the protein during sexual parasite development. Parasitology.

[84] Lippuner C (2018). RNA-Seq analysis during the life cycle of *Cryptosporidium parvum* reveals significant differential gene expression between proliferating stages in the intestine and infectious sporozoites. Int. J. Parasitol..

[85] Jonscher E, Erdbeer A, Günther M, Kurth M (2015). Two COWP-like cysteine rich proteins from *Eimeria nieschulzi* (coccidia, apicomplexa) are expressed during sporulation and involved in the sporocyst wall formation. Parasit. Vectors.

[86] Salman D (2017). Evaluation of novel oocyst wall protein candidates of *Toxoplasma gondii*. Parasitol. Int..

[87] Possenti A (2010). Molecular characterisation of a novel family of cysteine-rich proteins of *Toxoplasma gondii* and ultrastructural evidence of oocyst wall localisation. Int. J. Parasitol..

[88] Belli SI, Wallach MG, Luxford C, Davies MJ, Smith NC (2003). Roles of tyrosine-rich precursor glycoproteins and dityrosine- and 3,4-dihydroxyphenylalanine-mediated protein cross-linking in development of the oocyst wall in the coccidian parasite *Eimeria maxima*. Eukaryot. Cell.

[89] Fritz HM, Bowyer PW, Bogyo M, Conrad PA, Boothroyd JC (2012). Proteomic analysis of fractionated *Toxoplasma* oocysts reveals clues to their environmental resistance. PLoS ONE.

[90] Walker RA (2016). Discovery of a tyrosine-rich sporocyst wall protein in *Eimeria tenella*. Parasit. Vectors.

[91] Bandini G, Albuquerque-Wendt A, Hegermann J, Samuelson J, Routier FH (2019). Protein O- and C-Glycosylation pathways in *Toxoplasma gondii* and *Plasmodium falciparum*. Parasitology.

[92] Walker RA, Slapetova I, Slapeta J, Miller CM, Smith NC (2010). The glycosylation pathway of *Eimeria tenella* is upregulated during gametocyte development and may play a role in oocyst wall formation. Eukaryot. Cell.

[93] Mai K (2011). Peroxidase catalysed cross-linking of an intrinsically unstructured protein via dityrosine bonds in the oocyst wall of the apicomplexan parasite, *Eimeria maxima*. Int. J. Parasitol..

[94] Wiedmer S (2020). Correlative light and electron microscopy of wall formation in *Eimeria nieschulzi*. Parasitol. Res..

[95] Ferguson DJP, Belli SI, Smith NC, Wallach MG (2003). The development of the macrogamete and oocyst wall in *Eimeria maxima*: immuno-light and electron microscopy. Int. J. Parasitol..

[96] Wallach M (1992). Maternal immunization with gametocyte antigens as a means of providing protective immunity against *Eimeria maxima* in chickens. Infect. Immun..

[97] Ding J, Liu QQ-R, Han J-P, Qian W-F, Liu QQ-R (2012). Anti-recombinant gametocyte 56 protein IgY protected chickens from homologous coccidian infection. J. Integr. Agric..

[98] Wiedmer S (2017). Passive immunization with *Eimeria tenella* gametocyte antigen 56 (EtGAM56) specific antibodies and active immunization trial with the epitope containing peptide. Vet. Parasitol..

[99] Wallach MG, Ashash U, Michael A, Smith NC (2008). Field application of a subunit vaccine against an enteric protozoan disease. PLoS ONE.

[100] Jang SI (2010). *Eimeria maxima* recombinant Gam82 gametocyte antigen vaccine protects against coccidiosis and augments humoral and cell-mediated immunity. Vaccine.

[101] Venkatas J, Adeleke MA (2019). A review of *Eimeria* antigen identification for the development of novel anticoccidial vaccines. Parasitol. Res..

[102] Chichester JA (2018). Safety and immunogenicity of a plant-produced Pfs25 virus-like particle as a transmission blocking vaccine against malaria: a Phase 1 dose-escalation study in healthy adults. Vaccine.

[103] Coelho CH (2021). A human monoclonal antibody blocks malaria transmission and defines a highly conserved neutralizing epitope on gametes. Nat. Commun..

[104] Gwadz RW, Carter R, Green I (1979). Gamete vaccines and transmission-blocking immunity in malaria. Bull. World Health Organ..

[105] Lasonder E (2016). Integrated transcriptomic and proteomic analyses of *P. falciparum* gametocytes: molecular insight into sex-specific processes and translational repression. Nucleic Acids Res..

[106] Talman AM (2014). Proteomic analysis of the *Plasmodium* male gamete reveals the key role for glycolysis in flagellar motility. Malar. J..

[107] Ferguson DJP (2002). *Toxoplasma gondii* and sex: essential or optional extra?. Trends Parasitol..

[108] Tomasina R, Francia ME (2020). The Structural and Molecular Underpinnings of Gametogenesis in *Toxoplasma gondii*. Front. Cell. Infect. Microbiol..

[109] Larsen NC, Rasmussen KR, Healey MC (1991). Production and partial characterization of monoclonal antibodies specific for the gamonts of *Eimeria tenella*. J. Parasitol..

[110] Blagborough A, Sinden R (2009). *Plasmodium berghei* HAP2 induces strong malaria transmission-blocking immunity in vivo and in vitro. Vaccine.

[111] Qiu Y (2020). Evaluation of *Plasmodium vivax* HAP2 as a transmission-blocking vaccine candidate. Vaccine.

[112] Angrisano F (2017). Targeting the conserved fusion loop of HAP2 inhibits the transmission of *Plasmodium berghei* and *falciparum*. Cell Rep..

[113] Schwarz L, Worliczek HL, Winkler M, Joachim A (2014). Superinfection of sows with *Cystoisospora suis* ante partum leads to a milder course of cystoisosporosis in suckling piglets. Vet. Parasitol..

[114] Palmieri N (2017). The genome of the protozoan parasite *Cystoisospora suis* and a reverse vaccinology approach to identify vaccine candidates. Int. J. Parasitol..

[115] Warr, A. *et al.* An improved pig reference genome sequence to enable pig genetics and genomics research. *bioRxiv* 668921 (2019). 10.1101/668921 10.1093/gigascience/giaa051 PMC7448572 32543654

[116] Groenen MAM (2012). Analyses of pig genomes provide insight into porcine demography and evolution. Nature.

[117] Dobin A (2013). STAR: Ultrafast universal RNA-seq aligner. Bioinformatics.

[118] Andrews, S. No Title. *FastQC: a quality control tool for high throughput sequence data* (2010). Available at: http://www.bioinformatics.babraham.ac.uk/projects/fastqc.

[119] Okonechnikov K, Conesa A, García-Alcalde F (2016). Qualimap 2: advanced multi-sample quality control for high-throughput sequencing data. Bioinformatics.

[120] Wang L, Wang S, Li W (2012). RSeQC: quality control of RNA-seq experiments. Bioinformatics.

[121] Wang L (2016). Measure transcript integrity using RNA-seq data. BMC Bioinformatics.

[122] Liao Y, Smyth GK, Shi W (2014). featureCounts: an efficient general purpose program for assigning sequence reads to genomic features. Bioinformatics.

[123] R Core Team (2020). — European Environment Agency. Available at: https://www.eea.europa.eu/data-and-maps/indicators/oxygen-consuming-substances-in-rivers/r-development-core-team-2006. (Accessed: 26th November 2021)

[124] Hoffman GE, Schadt EE (2016). variancePartition: Interpreting drivers of variation in complex gene expression studies. BMC Bioinformatics.

[125] Hoffman GE, Roussos P (2021). Dream: powerful differential expression analysis for repeated measures designs. Bioinformatics.

[126] Benjamini Y, Hochberg Y (1995). Controlling the false discovery rate: a practical and powerful approach to multiple testing. J. R. Stat. Soc. Ser. B.

[127] Alexa, A. & Rahnenfuhrer, J. topGO: Enrichment Analysis for Gene Ontology. *R package version 2.40.0* (2020).

