# Supplementary figures and images for: The transcriptome from asexual to sexual in vitro development of Cystoisospora suis (Apicomplexa: Coccidia)
Source: Sci Rep. 2022 Apr 8;12:5972. doi: 10.1038/s41598-022-09714-8 (PMC8993856; doi:10.1038/s41598-022-09714-8)

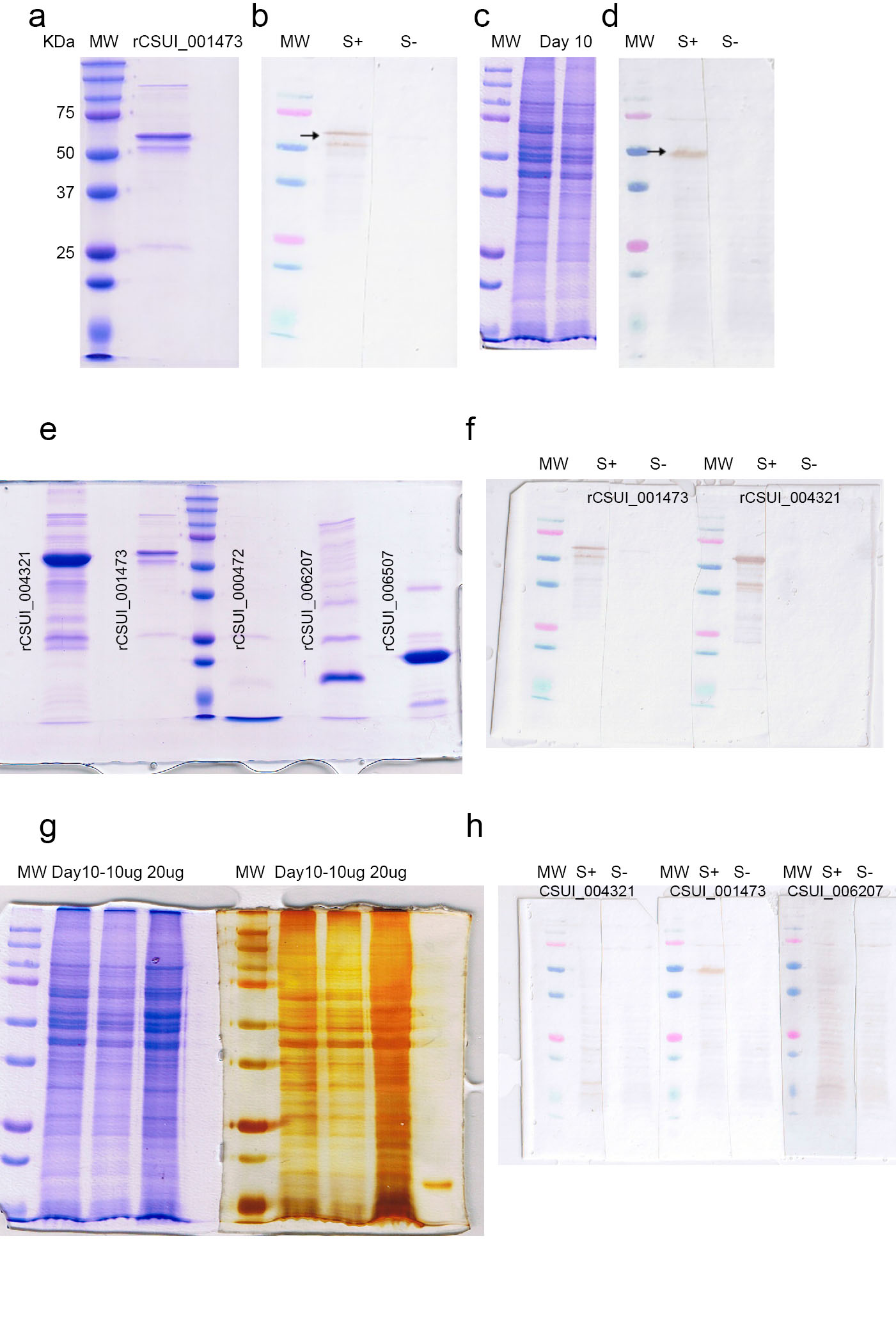

Supplement: Supplementary file 1 — Supplementary Information 1. [file 41598_2022_9714_MOESM1_ESM.jpg]
