# Supplementary material for: The transcriptome from asexual to sexual in vitro development of Cystoisospora suis (Apicomplexa: Coccidia)
Source: Sci Rep. 2022 Apr 8;12:5972. doi: 10.1038/s41598-022-09714-8 (PMC8993856; doi:10.1038/s41598-022-09714-8)
Supplement: Supplementary file 2 — Supplementary Information 2. [file 41598_2022_9714_MOESM2_ESM.docx]

**Figure-Table Supplementary Legends**

**Figure S1. Expression of rCSUI_001473 and Western blot analysis**. (**a**) Expression of rCSUI_001473. Lane M: molecular weight marker. A) Analysis of expressed recombinant protein by SDS-PAGE: protein lysates from cultures induced with 1 mM IPTG for 4 h; arrows indicate target protein bands of ~55 kDa. (**b**) Western blot of batch purified rCSUI_001473 probed with anti-rCSUI_001473 serum (S+) and pre-immunisation sera (S-). (**c** and **d**) SDS-PAGE and Western blot analyses of protein lysates of *C. suis* sexual stages from day 10 of culture probed with anti- rCSUI_001473 serum (S+) and pre-immunisation sera (S-). Arrows indicate the target protein bands of ~48 kDa. (**e** to **h**) originals SDS-PAGE gels and Western-blots.

**Table S1. Total paired reads mapped uniquely to *C. suis* genome.** Listed each RNASeq libraries of the seven biological replicates at each time point.

**Table S2. Differential expression of gene transcription in *C. suis*.** Transcript abundance and annotation of *C. suis* up- and downregulated transcripts cpmared by three time points of parasite cultivation. The log2 fold change, FDR- adjusted p-value, description and annotation are also listed.

**Table S3. Downregulated transcripts coding for proteins with either a known or putative role in sexual stages**. They are listed along with their transcript abundance (LogFC), annotation, comparison (downregulated transcripts (DT) in early sexual stages(2) compared to merozoites(1), DT12, late sexual stages (3) compared to merozoites (1), DT13, and late sexual stages (3) compared to early sexual stages(2), DT23)) and biological function.

**Table S4. Up- and downregulated transcripts coding for proteins with either a known or putative role in sexual commitment**. They are listed along with their transcript abundance (LogFC), annotation, comparison (upregulated (UT) and downregulated transcripts (DT) in early sexual stages(2) compared to merozoites(1), late sexual stages (3) compared to merozoites (1), and late sexual stages (3) compared to early sexual stages(2),) and biological function.

**Table S5. Upregulated transcripts coding for proteins with either a known or putative role in oocyst wall composition and surface**. They are listed along with their transcript abundance (LogFC), annotation, comparison (upregulated transcripts (UT) in early sexual stages(2) compared to merozoites(1), UT12, late sexual stages (3) compared to merozoites (1), UT13, and late sexual stages (3) compared to early sexual stages(2), UT23) and biological function.

**Table S6. Upregulated transcripts coding for proteins with either a known or putative role in microgamete biology**. They are listed along with their transcript abundance (LogFC), annotation, comparison (upregulated transcripts (UT) in early sexual stages(2) compared to merozoites(1), UT12, late sexual stages (3) compared to merozoites (1), UT13, and late sexual stages (3) compared to early sexual stages(2), UT23) and biological function.

**Table S7. Primers used in this work.**

**Supplemental file S1. Primers efficiency.**

**Supplemental file S2. Go term description**
