# Supplementary material for: The transcriptome from asexual to sexual in vitro development of Cystoisospora suis (Apicomplexa: Coccidia)
Source: Sci Rep. 2022 Apr 8;12:5972. doi: 10.1038/s41598-022-09714-8 (PMC8993856; doi:10.1038/s41598-022-09714-8)
Supplement: Supplementary file 3 — Supplementary Information 3. [file 41598_2022_9714_MOESM3_ESM.docx]

**Supplemental file S2. Go term description**

GO.ID Term Downregulated

GO:0006468 protein phosphorylation

GO:0006508 proteolysis

GO:0008643 carbohydrate transport

GO:0007018 microtubule-based movement

GO:0015074 DNA integration

GO:0051726 regulation of cell cycle

GO:0004672 protein kinase activity

GO:0005509 calcium ion binding

GO:0005215 transporter activity

GO:0005524 ATP binding

GO:0003777 microtubule motor activity

GO:0005328 neurotransmitter:sodium symporter activity

GO:0005576 extracellular region

GO:0016020 membrane

GO:0016021 integral component of membrane

GO:0030286 dynein complex

GO:0006355 regulation of transcription, DNA-templated

GO:0035556 intracellular signal transduction

GO:0003700 DNA-binding transcription factor activity

GO:0008233 peptidase activity

GO:0015293 symporter activity

GO:0061630 ubiquitin protein ligase activity

GO:0007165 signal transduction

GO:0055085 transmembrane transport

GO:0015693 magnesium ion transport

GO:0004114 3',5'-cyclic-nucleotide phosphodiesterase activity

GO:0046872 metal ion binding

GO:0022857 transmembrane transporter activity

GO:0015095 magnesium ion transmembrane transporter activity

GO:0004674 protein serine/threonine kinase activity

GO:0020037 heme binding

GO.ID Term

GO:1901605 alpha-amino acid metabolic process

GO:0006508 proteolysis

GO:0015074 DNA integration

GO:0051179 localization

GO:0044248 cellular catabolic process

GO:1901565 organonitrogen compound catabolic process

GO:0007018 microtubule-based movement

GO:0050660 flavin adenine dinucleotide binding

GO:0004252 serine-type endopeptidase activity

GO:0016614 oxidoreductase activity, acting on CH-OH group of donors

GO:0061630 ubiquitin protein ligase activity

GO:0043226 organelle

GO:0030688 preribosome, small subunit precursor

GO:0005576 extracellular region

GO:0007017 microtubule-based process

GO:0016052 carbohydrate catabolic process

GO:0044282 small molecule catabolic process

GO:0046173 polyol biosynthetic process

GO:0005996 monosaccharide metabolic process

GO:0009057 macromolecule catabolic process

GO:0044262 cellular carbohydrate metabolic process

GO:0006725 cellular aromatic compound metabolic process

GO:1901360 organic cyclic compound metabolic process

GO:0044260 cellular macromolecule metabolic process

GO:0005200 structural constituent of cytoskeleton

GO:0003777 microtubule motor activity

GO:0008017 microtubule binding

GO:0015631 tubulin binding

GO:0016758 transferase activity, transferring hexosyl groups

GO:0005874 microtubule

GO:0030286 dynein complex

GO:0046434 organophosphate catabolic process

GO:0043647 inositol phosphate metabolic process

GO:0045454 cell redox homeostasis

GO:0055114 oxidation-reduction process

GO:0009166 nucleotide catabolic process

GO:0009262 deoxyribonucleotide metabolic process

GO:0044272 sulfur compound biosynthetic process

GO:1901136 carbohydrate derivative catabolic process

GO:0006633 fatty acid biosynthetic process

GO:0008654 phospholipid biosynthetic process

GO:0046982 protein heterodimerization activity

GO:0003774 motor activity

GO:0016747 transferase activity, transferring groups other than amino-acyl groups

GO:0003690 double-stranded DNA binding

GO:0016620 oxidoreductase activity, acting on the aldehyde or oxo group of donors, NAD or NADP as acceptor

GO:0016829 lyase activity

GO:0016836 hydro-lyase activity

GO:0016667 oxidoreductase activity, acting on a sulfur group of donors

GO:0000786 nucleosome

GO:0005623 cell

GO:0005783 endoplasmic reticulum
