# Supplementary material for: The transcriptome from asexual to sexual in vitro development of Cystoisospora suis (Apicomplexa: Coccidia)
Source: Sci Rep. 2022 Apr 8;12:5972. doi: 10.1038/s41598-022-09714-8 (PMC8993856; doi:10.1038/s41598-022-09714-8)
Supplement: Supplementary file 7 — Supplementary Information 7. [file 41598_2022_9714_MOESM7_ESM.docx]

| Gene ID | logFC | FDR_adj_pval | Annotation | comparison | Function |  |
| --- | --- | --- | --- | --- | --- | --- |
|  |  |  |  |  |  |  |
| CSUI_008960 | -3.50 | 5,39E+02 | mic2-associated protein m2ap | DT12_DT23_DT13 | Host cell-attachment/Invasion |  |
| CSUI_006726 | -3.60 | 9,41E+05 | micronemal protein 8 | DT12_DT23_DT13 | Host cell-attachment/Invasion |  |
| CSUI_009604 | -1.23 | 0.008098 | microneme protein | DT12_DT13 | Host cell-attachment/Invasion |  |
| CSUI_002748 | -3.39 | 4,38E+02 | microneme protein | DT12_DT23_DT13 | Host cell-attachment/Invasion |  |
| CSUI_003520 | -2.23 | 3,05E+05 | microneme protein | DT12_DT23_DT13 | Host cell-attachment/Invasion |  |
| CSUI_001209 | -3.97 | 1,09E+04 | microneme protein 13 | DT12_DT23_DT13 | Host cell-attachment/Invasion |  |
| CSUI_001458 | -2.55 | 7,28E+05 | microneme protein 13 | DT12_DT23_DT13 | Host cell-attachment/Invasion |  |
| CSUI_003617 | -2.97 | 3,91E+04 | microneme protein 13 | DT23_DT13 | Host cell-attachment/Invasion |  |
| CSUI_002647 | -2.35 | 2,89E+05 | microneme protein 13 | DT23_DT13 | Host cell-attachment/Invasion |  |
| CSUI_000791 | -4.57 | 1,47E+04 | microneme protein 12 | DT12_DT23_DT13 | Host cell-attachment/Invasion |  |
| CSUI_010822 | -3.56 | 7,72E+02 | microneme protein 14 | DT12_DT23_DT13 | Host cell-attachment/Invasion |  |
| CSUI_003661 | -2.14 | 1,33E+04 | microneme protein 16 | DT23_DT13 | Host cell-attachment/Invasion |  |
| CSUI_010037 | -4.03 | 2,01E+03 | microneme protein 3 | DT12_DT23_DT13 | Host cell-attachment/Invasion |  |
| CSUI_006265 | -4.16 | 7,72E+02 | microneme protein 4 | DT12_DT23_DT13 | Host cell-attachment/Invasion |  |
| CSUI_008952 | -3.62 | 2,14E+05 | microneme protein 4 | DT12_DT23_DT13 | Host cell-attachment/Invasion |  |
| CSUI_002374 | -2.54 | 1,53E+02 | microneme protein 4 | DT12_DT23_DT13 | Host cell-attachment/Invasion |  |
| CSUI_010823 | -1.07 | 8,34E+07 | microneme protein 4 | DT13 | Host cell-attachment/Invasion |  |
| CSUI_006321 | -2.36 | 1,03E+05 | microneme protein 4 | DT23_DT13 | Host cell-attachment/Invasion |  |
| CSUI_000673 | -2.80 | 4,41E+07 | microneme protein 6 | DT12_DT23_DT13 | Host cell-attachment/Invasion |  |
| CSUI_001773 | -3.00 | 1,28E+06 | pan apple domain-containing protein | DT12_DT23_DT13 | Host cell-attachment/Invasion |  |
| CSUI_007850 | -4.57 | 1,58E+04 | pan domain-containing protein | DT12_DT23_DT13 | Host cell-attachment/Invasion |  |
| CSUI_001774 | -3.84 | 1,80E+03 | pan domain-containing protein | DT12_DT23_DT13 | Host cell-attachment/Invasion |  |
| CSUI_010224 | -3.76 | 7,67E+02 | pan domain-containing protein | DT12_DT23_DT13 | Host cell-attachment/Invasion |  |
| CSUI_004141 | -2.23 | 5,76E+04 | pan domain-containing protein | DT12_DT23_DT13 | Host cell-attachment/Invasion |  |
| CSUI_000216 | -1.27 | 0.000306 | pan domain-containing protein | DT13 | Host cell-attachment/Invasion |  |
| CSUI_007851 | -4.40 | 6,55E+03 | pan domain-containing protein | DT12_DT23_DT13 | Host cell-attachment/Invasion |  |
|  |  |  |  |  |  |  |
| CSUI_011194 | -1.62 | 0.000363 | rhoptry kinase family protein | DT12_DT13 | Invasion/Virulence |  |
| CSUI_005741 | -1.88 | 2,90E+06 | rhoptry kinase family protein rop11 (incomplete catalytic triad) | DT12_DT13 | Invasion/Virulence |  |
| CSUI_005979 | -1.61 | 2,16E+06 | rhoptry kinase family protein rop11 (incomplete catalytic triad) | DT12_DT13 | Invasion/Virulence |  |
| CSUI_005981 | -2.99 | 1,35E+06 | rhoptry kinase family protein rop11 (incomplete catalytic triad) | DT12_DT23_DT13 | Invasion/Virulence |  |
| CSUI_005840 | -1.09 | 6,59E+06 | rhoptry kinase family protein rop25 | DT23 | Invasion/Virulence |  |
| CSUI_004221 | -3.61 | 3,77E+05 | rhoptry kinase family protein rop28 | DT12_DT23_DT13 | Invasion/Virulence |  |
| CSUI_003834 | -3.43 | 4,37E+05 | rhoptry kinase family protein rop28 | DT12_DT23_DT13 | Invasion/Virulence |  |
| CSUI_007762 | -3.65 | 5,82E+04 | rhoptry kinase family protein rop32 | DT12_DT23_DT13 | Invasion/Virulence |  |
| CSUI_004303 | -1.04 | 1,47E+09 | rhoptry kinase family protein rop35 | DT13 | Invasion/Virulence |  |
| CSUI_010099 | -2.00 | 1,29E+06 | rhoptry kinase family protein rop35 | DT23_DT13 | Invasion/Virulence |  |
| CSUI_002921 | -1.50 | 2,35E+06 | rhoptry kinase family protein rop35 | DT23_DT13 | Invasion/Virulence |  |
| CSUI_002064 | -1.91 | 3,22E+07 | rhoptry kinase family protein rop37 (incomplete catalytic triad) | DT12_DT13 | Invasion/Virulence |  |
| CSUI_009292 | -1.48 | 2,51E+08 | rhoptry metalloprotease toxolysin tln1 | DT12_DT13 | Invasion/Virulence |  |
| CSUI_004021 | -1.85 | 3,43E+06 | rhoptry metalloprotease toxolysin tln1 | DT12_DT13 | Invasion/Virulence |  |
| CSUI_004256 | -1.09 | 6,57E+08 | rhoptry metalloprotease toxolysin tln1 | DT13 | Invasion/Virulence |  |
| CSUI_010184 | -2.13 | 3,11E+06 | rhoptry neck protein | DT12_DT13 | Invasion/Virulence |  |
| CSUI_007315 | -2.72 | 2,74E+05 | rhoptry neck protein | DT12_DT23_DT13 | Invasion/Virulence |  |
| CSUI_005876 | -1.24 | 4,31E+07 | rhoptry neck protein ron10 | DT12 | Invasion/Virulence |  |
| CSUI_001101 | -1.14 | 0.002013 | rhoptry neck protein ron2 | DT13 | Invasion/Virulence |  |
| CSUI_007149 | -4.07 | 1,49E+03 | rhoptry neck protein ron3 | DT12_DT23_DT13 | Invasion/Virulence |  |
| CSUI_004042 | -3.09 | 1,09E+06 | rhoptry neck protein ron4 | DT12_DT23_DT13 | Invasion/Virulence |  |
| CSUI_003545 | -1.69 | 8,93E+06 | rhoptry neck protein ron5 | DT12_DT13 | Invasion/Virulence |  |
| CSUI_002207 | -3.08 | 3,66E+04 | rhoptry neck protein ron6 | DT12_DT23_DT13 | Invasion/Virulence |  |
| CSUI_007069 | -2.57 | 6,63E+04 | rhoptry neck protein ron8 | DT12_DT13 | Invasion/Virulence |  |
| CSUI_000805 | -1.29 | 4,29E+09 | rhoptry protein | DT12_DT13 | Invasion/Virulence |  |
| CSUI_003286 | -1.06 | 0.000742 | rhoptry protein 5b | DT12 | Invasion/Virulence |  |
| CSUI_007217 | -2.77 | 1,22E+08 | rhoptry protein rop10 | DT12_DT23_DT13 | Invasion/Virulence |  |
| CSUI_002983 | -3.66 | 1,95E+04 | rhoptry protein rop12 | DT12_DT23_DT13 | Invasion/Virulence |  |
| CSUI_005437 | -1.83 | 6,74E+08 | rhoptry protein rop14 | DT23_DT13 | Invasion/Virulence |  |
| CSUI_001198 | -1.13 | 0.000224 | rhoptry protein rop15 | DT13 | Invasion/Virulence |  |
| CSUI_009576 | -3.09 | 2,81E+03 | rhoptry protein rop17 | DT12_DT23_DT13 | Invasion/Virulence |  |
| CSUI_009095 | -3.89 | 1,99E+04 | rhoptry protein rop6 | DT12_DT23_DT13 | Invasion/Virulence |  |
|  |  |  |  |  |  |  |
| CSUI_001322 | -1.40 | 0.002454 | dense granule protein gra12 | DT12_DT13 | Biogenesis and maturation of the PV |  |
| CSUI_010884 | -1.63 | 1,92E+06 | dense granule protein gra12 | DT13 | Biogenesis and maturation of the PV |  |
|  |  |  |  |  |  |  |
| CSUI_001098 | -1.66 | 3,48E+06 | apical membrane antigen 1 domain-containing protein (AMA1) | DT23_DT13 | Moving junction |  |
| CSUI_004137 | -2.55 | 6,55E+03 | sporozoite protein with an altered thrombospondin repeat spatr | DT12_DT23_DT13 | Moving junction |  |
| CSUI_003734 | -1.33 | 1,22E+05 | apical cap protein 1 | DT13 | Moving junction |  |
|  |  |  |  |  |  |  |
| CSUI_001007 | -2.06 | 8,91E+03 | glideosome-associated protein with multiple-membrane spans gapm2b | DT12_DT23_DT13 | Gliding motility |  |
| CSUI_000298 | -1.19 | 3,68E+09 | glideosome-associated protein with multiple-membrane spans gapm3 | DT13 | Gliding motility |  |
| CSUI_001504 | -1.96 | 4,17E+06 | gliding-associated protein gap70 | DT23_DT13 | Gliding motility |  |
| CSUI_006251 | -1.83 | 5,95E+05 | gap40 protein | DT23_DT13 | Gliding motility |  |
|  |  |  |  |  |  |  |
| CSUI_008181 | -2.37 | 9,03E+04 | dynein heavy chain | DT12_DT13 | Microtubule |  |
| CSUI_003685 | -1.19 | 0.000139 | dynein light intermediate | DT12_DT13 | Microtubule |  |
| CSUI_000001 | -1.74 | 3,69E+06 | kinesin heavy | DT13 | Microtubule |  |
| CSUI_006136 | -2.05 | 1,20E+08 | kinesin motor domain-containing protein | DT12_DT13 | Microtubule |  |
| CSUI_005377 | -2.01 | 2,25E+09 | myosin c | DT12_DT13 | Microtubule |  |
| CSUI_000307 | -1.74 | 1,15E+08 | myosin h | DT23_DT13 | Microtubule |  |
| CSUI_004205 | -1.79 | 6,52E+05 | myosin head (motor domain) domain-containing protein | DT12_DT13 | Microtubule |  |
| CSUI_003244 | -2.20 | 7,04E+06 | myosin i | DT23_DT13 | Microtubule |  |
| CSUI_008617 | -2.03 | 1,95E+07 | myosin light chain | DT12_DT13 | Microtubule |  |
| CSUI_000910 | -1.02 | 6,19E+08 | myosin light chain | DT13 | Microtubule |  |
| CSUI_006043 | -1.67 | 0.000194 | myosin light chain 2 | DT12_DT13 | Microtubule |  |
| CSUI_003181 | -1.24 | 2,96E+07 | myosin light chain mlc1 | DT13 | Microtubule |  |
| CSUI_001614 | -1.18 | 0.000137 | phosphatidylinositol 3- and 4-kinase | DT13 | Microtubule |  |
| CSUI_009668 | -1.25 | 1,91E+07 | protein kinase (incomplete catalytic triad) | DT12_DT13 | Microtubule |  |
| CSUI_005294 | -1.50 | 1,36E+08 | tubulin gtpase domain-containing protein | DT13 | Microtubule |  |
| CSUI_005558 | -2.74 | 7,11E+05 | tubulin-tyrosine ligase family protein | DT12_DT23_DT13 | Microtubule |  |
| CSUI_001972 | -2.14 | 1,48E+05 | tubulin-tyrosine ligase family protein | DT12_DT23_DT13 | Microtubule |  |
| CSUI_011059 | -1.81 | 1,41E+09 | tubulin-tyrosine ligase family protein | DT23_DT13 | Microtubule |  |
| CSUI_003468 | -2.28 | 1,58E+06 | microtubule associated protein spm2 | DT23_DT13 | Microtubule |  |
| CSUI_005135 | -1.95 | 8,38E+07 | actin-related protein arp1 | DT12_DT13 | Microtubule |  |
| CSUI_001167 | -2.00 | 2,42E+07 | formin frm1 | DT23_DT13 | Microtubule |  |
| CSUI_010009 | -1.26 | 0.000213 | formin frm2 | DT23 | Microtubule |  |
|  |  |  |  |  |  |  |
| CSUI_003486 | -2.93 | 2,82E+06 | alveolin domain containing intermediate filament imc6 | DT23_DT13 | IMC complex |  |
| CSUI_010652 | -2.59 | 2,16E+04 | alveolin domain containing intermediate filament imc7 | DT23_DT13 | IMC complex |  |
| CSUI_004655 | -2.23 | 1,13E+06 | alveolin domain containing intermediate filament imc8 | DT12_DT13 | IMC complex |  |
| CSUI_009009 | -2.24 | 0.000211 | inner membrane complex protein | DT23_DT13 | IMC complex |  |
| CSUI_009164 | -1.43 | 1,09E+08 | inner membrane complex protein 18 | DT13 | IMC complex |  |
| CSUI_000889 | -2.23 | 1,43E+05 | inner membrane complex protein 19 | DT23_DT13 | IMC complex |  |
| CSUI_000302 | -2.78 | 1,81E+04 | inner membrane complex protein 20 | DT23_DT13 | IMC complex |  |
| CSUI_004345 | -2.87 | 2,64E+02 | inner membrane complex protein 22 | DT23_DT13 | IMC complex |  |
| CSUI_002278 | -2.65 | 8,56E+04 | inner membrane complex protein 24 | DT12_DT23_DT13 | IMC complex |  |
| CSUI_006156 | -1.57 | 0.002546 | imc sub-compartment protein isp1 | DT12_DT13 | IMC complex |  |
| CSUI_001891 | -2.80 | 3,25E+05 | imc sub-compartment protein isp2 | DT23_DT13 | IMC complex |  |
| CSUI_005485 | -1.68 | 0.000232 | imc sub-compartment protein isp3 | DT12_DT13 | IMC complex |  |
| CSUI_002780 | -2.01 | 3,07E+09 | imc subcompartment protein isp4 | DT23_DT13 | IMC complex |  |
| CSUI_004027 | -1.49 | 0.001392 | imc-associated protein 1 | DT12_DT13 | IMC complex |  |
|  |  |  |  |  |  |  |
| CSUI_001629 | -1.15 | 4,38E+05 | agc kinase | DT13 | phosphorylation |  |
| CSUI_002874 | -1.02 | 0.029050 | calcium-dependent protein kinase cdpk4a | DT12 | phosphorylation |  |
| CSUI_001668 | -2.25 | 1,77E+02 | calcium-dependent protein kinase cdpk5 | DT12_DT23_DT13 | phosphorylation |  |
| CSUI_006563 | -1.37 | 0.013371 | calcium-dependent protein kinase cdpk9 | DT23_DT13 | phosphorylation |  |
| CSUI_002319 | -1.56 | 1,20E+07 | cam cdpk | DT13 | phosphorylation |  |
| CSUI_010130 | -1.85 | 9,51E+06 | camk camkl protein kinase | DT12_DT13 | phosphorylation |  |
| CSUI_000151 | -1.15 | 5,23E+07 | camk cdpk protein kinase | DT13 | phosphorylation |  |
| CSUI_003182 | -1.42 | 2,00E+09 | cmgc kinase | DT13 | phosphorylation |  |
| CSUI_002148 | -1.15 | 4,04E+06 | cmgc mapk family mapk-1 | DT13 | phosphorylation |  |
| CSUI_007210 | -1.71 | 4,24E+07 | calcium binding egf domain-containing protein | DT12_DT13 | phosphorylation |  |
| CSUI_000979 | -3.68 | 1,53E+02 | calcium binding egf domain-containing protein | DT12_DT23_DT13 | phosphorylation |  |
| CSUI_000982 | -1.96 | 1,50E+05 | calcium binding egf domain-containing protein | DT23_DT13 | phosphorylation |  |
| CSUI_010907 | -2.26 | 0.000215 | calcium-dependent protein kinase cdpk3 | DT12_DT13 | phosphorylation |  |
| CSUI_003507 | -1.11 | 7,19E+04 | calmodulin | DT23 | phosphorylation |  |
| CSUI_000865 | -2.06 | 2,03E+07 | camp-dependent protein kinase regulatory | DT23_DT13 | phosphorylation |  |
| CSUI_008652 | -1.00 | 0.009193 | eif2 kinase if2k-c | DT12 | phosphorylation |  |
| CSUI_005541 | -2.12 | 9,40E+05 | hypothetical protein | DT23_DT13 | phosphorylation |  |
| CSUI_000671 | -1.34 | 2,59E+07 | pik3r4 kinase-related protein | DT13 | phosphorylation |  |
| CSUI_005927 | -4.34 | 2,43E+04 | protein kinase | DT12_DT23_DT13 | phosphorylation |  |
| CSUI_008664 | -2.03 | 3,32E+04 | protein kinase | DT23_DT13 | phosphorylation |  |
| CSUI_000114 | -3.13 | 7,52E+05 | protein kinase (incomplete catalytic triad) | DT12_DT23_DT13 | phosphorylation |  |
| CSUI_011418 | -2.57 | 1,30E+08 | protein kinase (incomplete catalytic triad) | DT12_DT23_DT13 | phosphorylation |  |
| CSUI_011530 | -1.48 | 7,94E+08 | protein kinase domain protein | DT12 | phosphorylation |  |
| CSUI_003792 | -1.45 | 0.000113 | protein kinase domain-containing protein | DT23 | phosphorylation |  |
| CSUI_008282 | -2.28 | 3,53E+04 | 3 5 -cyclic nucleotide phosphodiesterase domain-containing protein | DT12_DT13 | signal transduction |  |
| CSUI_002875 | -1.34 | 4,37E+08 | 3 5 -cyclic nucleotide phosphodiesterase domain-containing protein | DT13 | signal transduction |  |
| CSUI_000710 | -1.23 | 3,38E+03 | 3 5 -cyclic nucleotide phosphodiesterase domain-containing protein | DT13 | signal transduction |  |
| CSUI_010693 | -1.05 | 1,45E+09 | 3 5 -cyclic nucleotide phosphodiesterase domain-containing protein | DT13 | signal transduction |  |
| CSUI_003602 | -1.01 | 4,77E+08 | 3 5 -cyclic nucleotide phosphodiesterase domain-containing protein | DT13 | signal transduction |  |
| CSUI_003343 | -1.78 | 1,74E+05 | 3 5 -cyclic nucleotide phosphodiesterase domain-containing protein | DT23_DT13 | signal transduction |  |
| CSUI_005822 | -1.40 | 2,62E+03 | diacylglycerol kinase | DT13 | signal transduction |  |
| CSUI_002494 | -1.68 | 1,07E+09 | doublecortin | DT23_DT13 | signal transduction |  |
| CSUI_001031 | -1.75 | 0.002118 | guanylyl cyclase | DT12_DT13 | signal transduction |  |
| CSUI_003554 | -1.39 | 2,41E+08 | guanylyl cyclase | DT12_DT13 | signal transduction |  |
| CSUI_008777 | -1.90 | 4,36E+05 | guanylyl cyclase | DT23_DT13 | signal transduction |  |
| CSUI_002537 | -1.97 | 4,74E+05 | hypothetical protein | DT23_DT13 | signal transduction |  |
| CSUI_010828 | -1.57 | 0.000255 | 3 --cyclic-nucleotide | DT12_DT13 | signal transduction |  |
| CSUI_009488 | -1.37 | 0.000154 | armadillo interacting protein | DT13 | signal transduction |  |
| CSUI_004337 | -1.03 | 3,87E+08 | c2 domain protein | DT13 | signal transduction |  |
| CSUI_007335 | -1.29 | 0.024262 | c2 domain-containing protein | DT12 | signal transduction |  |
| CSUI_000831 | -1.48 | 4,77E+09 | c2 domain-containing protein | DT12_DT13 | signal transduction |  |
| CSUI_010773 | -1.63 | 5,67E+07 | coronin | DT13 | signal transduction |  |
| CSUI_000690 | -1.11 | 5,12E+09 | guanylate binding | DT12 | signal transduction |  |
|  |  |  |  |  |  |  |
| CSUI_009013 | -2.87 | 3,47E+08 | srs domain-containing protein | DT12_DT13 | Adhesion/Invasion |  |
| CSUI_001532 | -2.14 | 8,66E+07 | srs domain-containing protein | DT12_DT13 | Adhesion/Invasion |  |
| CSUI_006047 | -2.10 | 0.000612 | srs domain-containing protein | DT12_DT13 | Adhesion/Invasion |  |
| CSUI_006281 | -1.89 | 2,37E+08 | srs domain-containing protein | DT12_DT13 | Adhesion/Invasion |  |
| CSUI_010846 | -3.84 | 2,33E+03 | srs domain-containing protein | DT12_DT23_DT13 | Adhesion/Invasion |  |
| CSUI_010847 | -3.76 | 1,54E+04 | srs domain-containing protein | DT12_DT23_DT13 | Adhesion/Invasion |  |
| CSUI_010845 | -3.36 | 3,08E+04 | srs domain-containing protein | DT12_DT23_DT13 | Adhesion/Invasion |  |
| CSUI_009012 | -3.05 | 1,54E+05 | srs domain-containing protein | DT12_DT23_DT13 | Adhesion/Invasion |  |
| CSUI_002407 | -3.05 | 3,75E+04 | srs domain-containing protein | DT12_DT23_DT13 | Adhesion/Invasion |  |
| CSUI_010203 | -3.04 | 1,09E+04 | srs domain-containing protein | DT12_DT23_DT13 | Adhesion/Invasion |  |
| CSUI_005990 | -2.85 | 3,76E+06 | srs domain-containing protein | DT12_DT23_DT13 | Adhesion/Invasion |  |
| CSUI_009055 | -2.83 | 1,90E+06 | srs domain-containing protein | DT12_DT23_DT13 | Adhesion/Invasion |  |
| CSUI_008559 | -2.43 | 2,15E+07 | srs domain-containing protein | DT12_DT23_DT13 | Adhesion/Invasion |  |
| CSUI_010529 | -1.58 | 1,79E+08 | srs domain-containing protein | DT13 | Adhesion/Invasion |  |
| CSUI_003788 | -1.49 | 4,45E+06 | srs domain-containing protein | DT13 | Adhesion/Invasion |  |
| CSUI_009427 | -1.45 | 6,78E+08 | srs domain-containing protein | DT13 | Adhesion/Invasion |  |
| CSUI_009011 | -1.32 | 2,50E+09 | srs domain-containing protein | DT13 | Adhesion/Invasion |  |
| CSUI_009424 | -1.27 | 7,62E+06 | srs domain-containing protein | DT13 | Adhesion/Invasion |  |
| CSUI_006282 | -1.19 | 1,89E+08 | srs domain-containing protein | DT13 | Adhesion/Invasion |  |
| CSUI_003818 | -1.06 | 2,16E+07 | srs domain-containing protein | DT13 | Adhesion/Invasion |  |
| CSUI_008686 | -2.42 | 3,84E+08 | srs domain-containing protein | DT23_DT13 | Adhesion/Invasion |  |
| CSUI_005469 | -2.39 | 2,76E+05 | srs domain-containing protein | DT23_DT13 | Adhesion/Invasion |  |
| CSUI_003351 | -2.06 | 0.000124 | srs domain-containing protein | DT23_DT13 | Adhesion/Invasion |  |
| CSUI_002409 | -1.97 | 1,14E+08 | srs domain-containing protein | DT23_DT13 | Adhesion/Invasion |  |
| CSUI_002410 | -1.92 | 8,52E+08 | srs domain-containing protein | DT23_DT13 | Adhesion/Invasion |  |
| CSUI_009947 | -1.88 | 2,89E+07 | srs domain-containing protein | DT23_DT13 | Adhesion/Invasion |  |
| CSUI_009426 | -1.29 | 1,07E+09 | srs domain-containing protein | DT23_DT13 | Adhesion/Invasion |  |
| CSUI_009631 | -1.43 | 6,99E+06 | sag-related sequence srs11 | DT13 | Adhesion/Invasion |  |
| CSUI_007387 | -2.00 | 1,08E+07 | sag-related sequence srs11 | DT23_DT13 | Adhesion/Invasion |  |
| CSUI_000322 | -1.88 | 2,03E+04 | sag-related sequence srs13 | DT23_DT13 | Adhesion/Invasion |  |
| CSUI_006555 | -1.97 | 1,71E+09 | sag-related sequence srs17a | DT12_DT13 | Adhesion/Invasion |  |
| CSUI_003092 | -1.51 | 8,01E+06 | sag-related sequence srs17a | DT13 | Adhesion/Invasion |  |
| CSUI_005568 | -3.27 | 6,60E+03 | sag-related sequence srs17b | DT12_DT23_DT13 | Adhesion/Invasion |  |
| CSUI_005950 | -1.16 | 0.001627 | sag-related sequence srs20c | DT13 | Adhesion/Invasion |  |
| CSUI_009618 | -2.22 | 1,40E+05 | sag-related sequence srs22c | DT23_DT13 | Adhesion/Invasion |  |
| CSUI_004444 | -1.54 | 5,57E+07 | sag-related sequence srs26i | DT13 | Adhesion/Invasion |  |
| CSUI_004246 | -3.46 | 3,74E+03 | sag-related sequence srs26j | DT12_DT23_DT13 | Adhesion/Invasion |  |
| CSUI_005473 | -1.47 | 1,89E+09 | sag-related sequence srs28 | DT13 | Adhesion/Invasion |  |
| CSUI_009059 | -2.57 | 6,79E+07 | sag-related sequence srs30d | DT23_DT13 | Adhesion/Invasion |  |
| CSUI_002341 | -2.97 | 1,06E+05 | sag-related sequence srs42 | DT12_DT23_DT13 | Adhesion/Invasion |  |
| CSUI_007679 | -1.33 | 9,17E+07 | sag-related sequence srs53a | DT13 | Adhesion/Invasion |  |
| CSUI_007477 | -1.76 | 3,73E+07 | sag-related sequence srs53c | DT13 | Adhesion/Invasion |  |
| CSUI_000034 | -1.33 | 9,45E+06 | sag-related sequence srs53c | DT13 | Adhesion/Invasion |  |
| CSUI_002641 | -2.54 | 9,04E+06 | sag-related sequence srs53c | DT23_DT13 | Adhesion/Invasion |  |
| CSUI_010479 | -2.88 | 3,23E+09 | sag-related sequence srs53f | DT12_DT23_DT13 | Adhesion/Invasion |  |
| CSUI_010484 | -2.52 | 7,22E+07 | sag-related sequence srs53f | DT12_DT23_DT13 | Adhesion/Invasion |  |
| CSUI_011516 | -1.07 | 0.009748 | sag-related sequence srs53f | DT13 | Adhesion/Invasion |  |
| CSUI_007678 | -2.90 | 2,25E+07 | sag-related sequence srs53f | DT23_DT13 | Adhesion/Invasion |  |
| CSUI_007474 | -2.52 | 2,52E+07 | sag-related sequence srs53f | DT23_DT13 | Adhesion/Invasion |  |
| CSUI_011314 | -2.19 | 1,77E+09 | sag-related sequence srs53f | DT23_DT13 | Adhesion/Invasion |  |
| CSUI_010578 | -1.92 | 1,57E+05 | sag-related sequence srs57 | DT23_DT13 | Adhesion/Invasion |  |
| CSUI_003814 | -1.29 | 0.006109 | surface antigen | DT23_DT13 | Adhesion/Invasion |  |
| CSUI_003681 | -1.37 | 1,68E+08 | surface antigen 2 | DT13 | Adhesion/Invasion |  |
|  |  |  |  |  |  |  |

Downregulated transcripts coding for proteins with either a known or putative role in invasion are listed along with their transcript abundance (LogFC), annotation and biological function.
