# Supplementary material for: The transcriptome from asexual to sexual in vitro development of Cystoisospora suis (Apicomplexa: Coccidia)
Source: Sci Rep. 2022 Apr 8;12:5972. doi: 10.1038/s41598-022-09714-8 (PMC8993856; doi:10.1038/s41598-022-09714-8)
Supplement: Supplementary file 8 — Supplementary Information 8. [file 41598_2022_9714_MOESM8_ESM.docx]

| Gene ID | logFC | FDR_adj_pval | Annotation | comparison | Function |
| --- | --- | --- | --- | --- | --- |
|  |  |  |  |  |  |
| CSUI_004230 | 1.28 | 7,24E+09 | ap2 domain transcription factor ap2iii-4 | UT23_UT13 | DNA binding proteins |
| CSUI_000155 | 1.47 | 4,38E+05 | ap2 domain transcription factor ap2ix-1 | UT12_UT13 | DNA binding proteins |
| CSUI_000476 | 1.15 | 0.00801 | ap2 domain transcription factor ap2v-2 | UT12 | DNA binding proteins |
| CSUI_001971 | 1.26 | 1,12E+08 | ap2 domain transcription factor ap2vi-3 | UT13 | DNA binding proteins |
| CSUI_000083 | 1.33 | 4,59E+04 | ap2 domain transcription factor ap2viii-5 | UT13 | DNA binding proteins |
| CSUI_009556 | 2.01 | 3,44E+09 | ap2 domain transcription factor ap2x-10 | UT23_UT13 | DNA binding proteins |
| CSUI_010124 | 1.06 | 0.005544 | ap2 domain transcription factor ap2x-8 | UT12 | DNA binding proteins |
|  |  |  |  |  |  |
| CSUI_004043 | -1.23 | 0.003102 | ap2 domain transcription factor ap2iii-2 | DT12_DT13 | Regulation of transcription |
| CSUI_003766 | -1.23 | 1,28E+06 | ap2 domain transcription factor ap2iv-2 | DT23_DT13 | Regulation of transcription |
| CSUI_000699 | -3.10 | 3,12E+05 | ap2 domain transcription factor ap2iv-4 | DT12_DT23_DT13 | Regulation of transcription |
| CSUI_003667 | -1.36 | 0.003078 | ap2 domain transcription factor ap2ix-5 | DT13 | Regulation of transcription |
| CSUI_002302 | -1.40 | 4,54E+09 | ap2 domain transcription factor ap2ix-7 | DT13 | Regulation of transcription |
| CSUI_004108 | -1.32 | 1,84E+07 | ap2 domain transcription factor ap2viia | DT23_DT13 | Regulation of transcription |
| CSUI_006213 | -4.16 | 5,46E+02 | ap2 domain transcription factor ap2viia-1 | DT12_DT23_DT13 | Regulation of transcription |
| CSUI_009186 | -2.09 | 5,23E+06 | ap2 domain transcription factor ap2viia-4 | DT23_DT13 | Regulation of transcription |
| CSUI_004491 | -1.40 | 0.01184 | ap2 domain transcription factor ap2viia-4 | DT12_DT13 | Regulation of transcription |
| CSUI_000651 | -1.67 | 1,03E+06 | ap2 domain transcription factor ap2viia-6 | DT23_DT13 | Regulation of transcription |
| CSUI_000127 | -1.44 | 0.000186 | ap2 domain transcription factor ap2viia-7 | DT23 | Regulation of transcription |
| CSUI_003191 | -1.12 | 6,28E+07 | ap2 domain transcription factor ap2viib-3 | DT13 | Regulation of transcription |
| CSUI_006100 | -1.07 | 1,41E+07 | ap2 domain transcription factor ap2viii-3 | DT13 | Regulation of transcription |
| CSUI_009147 | -2.18 | 7,58E+07 | ap2 domain transcription factor ap2viii-6 | DT12_DT23_DT13 | Regulation of transcription |
| CSUI_001754 | -2.66 | 5,43E+02 | ap2 domain transcription factor ap2viii-7 | DT23_DT13 | Regulation of transcription |
| CSUI_004414 | -1.34 | 3,38E+08 | ap2 domain transcription factor ap2x-3 | DT23 | Regulation of transcription |
| CSUI_006526 | -1.15 | 0.000215 | ap2 domain transcription factor ap2x-8 | DT13 | Regulation of transcription |
| CSUI_010124 | -1.21 | 3,33E+09 | ap2 domain transcription factor ap2x-8 | DT23 | Regulation of transcription |
| CSUI_000465 | -1.96 | 1,89E+05 | ap2 domain transcription factor ap2xi-1 | DT23_DT13 | Regulation of transcription |
| CSUI_010170 | -1.24 | 2,68E+09 | ap2 domain transcription factor ap2xii-4 | DT23 | Regulation of transcription |
| CSUI_003177 | -1.68 | 4,70E+08 | ap2 domain transcription factor ap2xii-8 | DT13 | Regulation of transcription |
| CSUI_007189 | -3.22 | 1,91E+06 | ap2 domain transcription factor ap2xii-9 | DT12_DT23_DT13 | Regulation of transcription, |
|  |  |  |  |  |  |
| CSUI_003805 | -1.20 | 0.0155638 | cyclin 4 | DT12_DT13 | Regulation of transcription |
| CSUI_008076 | -1.09 | 0.0132182 | not1 amine-terminal ccr4-not complex component | DT12_DT13 | Regulation of transcription |
| CSUI_002917 | -1.30 | 8,28E+09 | dna polymerase phi subunit | DT13 | Regulation of transcription |
| CSUI_009086 | -1.08 | 0.0068712 | tetratricopeptide repeat-containing protein | DT13 | Regulation of transcription |
| CSUI_010185 | -2.49 | 3,87E+04 | transcription factor with ap2 domain | DT23_DT13 | Regulation of transcription |
|  |  |  |  |  |  |
| CSUI_005258 | -1.12 | 0.0008381 | n-terminal domain-containing protein | DT13 | Regulation cell cycle |
| CSUI_010431 | -1.52 | 0.0009810 | cyclin2 related protein | DT12_DT13 | Regulation cell cycle |
| CSUI_000308 | -1.21 | 5,64E+09 | fatc domain-containing protein | DT13 | Regulation cell cycle |
|  |  |  |  |  |  |
| CSUI_003099 | 3.75 | 4,45E+02 | cmgc ck2 family-NIMA2 | UT12_UT23_UT13 | Regulation cell cycle |
|  |  |  |  |  |  |
| CSUI_006334 | -1.39 | 4,97E+09 | ppg3 (proteophosphoglycan) | DT13 | Bradyzoite cyst wall |
| CSUI_006268 | -1.44 | 0.000181 | ppg3 (proteophosphoglycan) | DT23_DT13 | Bradyzoite cyst wall |
| CSUI_002770 | -1.96 | 7,96E+08 | proteophosphoglycan protein ppg4 | DT23_DT13 | Bradyzoite cyst wall |
| CSUI_001601 | -2.20 | 3,08E+05 | proteophosphoglycan related | DT23_DT13 | Bradyzoite cyst wall |
| CSUI_000464 | -1.34 | 2,29E+08 | proteophosphoglycan related | DT12_DT13 | Bradyzoite cyst wall |
| CSUI_000106 | -1.69 | 6,41E+04 | proteophosphoglycan related protein | DT23_DT13 | Bradyzoite cyst wall |
| CSUI_006681 | -1.16 | 0.007466 | proteophosphoglycan related protein | DT13 | Bradyzoite cyst wall |
| CSUI_003656 | -2.21 | 4,61E+04 | heat shock protein hsp29 | DT23_DT13 | Bradyzoite antigen |
| CSUI_003013 | -3.35 | 4,15E+02 | hypothetical protein | DT12_DT23_DT13 | Bradyzoite antigen |
| CSUI_001328 | -1.74 | 2,45E+09 | myb family dna-binding domain-containing protein | DT13 | Bradyzoite antigen |
| CSUI_003154 | -1.04 | 5,56E+07 | serpin (serine proteinase inhibitor) superfamily protein | DT13 | Bradyzoite antigen |
| CSUI_001507 | -2.41 | 5,62E+05 | heat shock protein | DT23_DT13 | Bradyzoite antigen |
|  |  |  |  |  |  |
