# Supplementary material for: The transcriptome from asexual to sexual in vitro development of Cystoisospora suis (Apicomplexa: Coccidia)
Source: Sci Rep. 2022 Apr 8;12:5972. doi: 10.1038/s41598-022-09714-8 (PMC8993856; doi:10.1038/s41598-022-09714-8)
Supplement: Supplementary file 9 — Supplementary Information 9. [file 41598_2022_9714_MOESM9_ESM.docx]

| Gene ID | logFC | FDR_adj_pval | Annotation | comparison | Function |
| --- | --- | --- | --- | --- | --- |
|  |  |  |  |  |  |
| CSUI_008806 | 4.62 | 1,47E+04 | oocyst wall protein | UT12_UT23_UT13 | Oocyst wall |
| CSUI_006207 | 1.75 | 1,04E+08 | oocyst wall protein | UT23_UT13 | Oocyst Wall |
| CSUI_002027 | 5.47 | 1,88E+04 | toxoplasma gondii family a protein | UT12_UT23_UT13 | Oocyst Wall |
| CSUI_006655 | 1.78 | 9,10E+06 | toxoplasma gondii family a protein | UT12_UT13 | Oocyst Wall |
| CSUI_010157 | 4.32 | 4,37E+05 | toxoplasma gondii family a protein | UT12_UT23_UT13 | Oocyst Wall |
| CSUI_003908 | 3.39 | 2,68E+06 | toxoplasma gondii family d protein | UT12_UT13 | Oocyst Wall |
| CSUI_004489 | 3.29 | 2,89E+05 | toxoplasma gondii family d protein | UT12_UT13 | Oocyst Wall |
| CSUI_004212 | 2.21 | 1,92E+07 | toxoplasma gondii family d protein | UT23_UT13 | Oocyst Wall |
| CSUI_009196 | 1.67 | 2,45E+09 | toxoplasma gondii family d protein | UT13 | Oocyst Wall |
| CSUI_000190 | 5.84 | 3,56E+03 | hypothetical protein-TyRP | UT12_UT23_UT13 | Oocyst Wall |
| CSUI_001473 | 2.77 | 9,93E+05 | hypothetical protein-TyRP | UT12_UT13 | Oocyst Wall |
| CSUI_001475 | 3.46 | 1,07E+05 | hypothetical protein-TyRP | UT12_UT13 | Oocyst Wall |
|  |  |  |  |  |  |
| CSUI_002430 | 1.29 | 7,54E+09 | dolichol-phosphate mannosyltransferase subunit 1 | UT13 | Glycosylation |
| CSUI_008811 | 1.26 | 6,09E+07 | dolichyl-diphosphooligosaccharide--protein glycosyltransferase | UT13 | Glycosylation |
| CSUI_000045 | 1.04 | 1,05E+04 | dolichyl-diphosphooligosaccharide--protein glycosyltransferase subunit dad1 | UT13 | Glycosylation |
| CSUI_005659 | 1.24 | 1,42E+08 | glycosyl family 31 protein | UT13 | Glycosylation |
| CSUI_003717 | 1.05 | 9,63E+09 | glycosyl transferase family 8 protein | UT13 | Glycosylation |
| CSUI_011212 | 1.58 | 2,84E+03 | glycosylphosphatidylinositol anchor attachment protein 1 | UT12_UT13 | Glycosylation |
| CSUI_004193 | 2.76 | 1,20E+07 | glycosyltransferase family 17 protein | UT12_UT23_UT13 | Glycosylation |
| CSUI_009433 | 1.12 | 5,49E+09 | glycosyltransferase family protein | UT13 | Glycosylation |
| CSUI_010916 | 1.11 | 2,12E+09 | mannosyltransferase | UT13 | Glycosylation |
| CSUI_004231 | 1.02 | 1,91E+07 | phosphatidylinositol n-acetylglucosaminyltransferase | UT13 | Glycosylation |
| CSUI_000082 | 4.30 | 2,83E+02 | 4-alpha-glucanotransferase | UT12_UT23_UT13 | Glycosylation |
|  |  |  |  |  |  |
| CSUI_004757 | 1.38 | 7,64E+03 | zinc finger in n-recognin protein | UT12 | Proteolysis |
| CSUI_000670 | 1.00 | 2,87E+09 | x-pro dipeptidyl-peptidase (s15 family) | UT13 | Proteolysis |
| CSUI_003678 | 1.02 | 1,60E+07 | Ubiquitin-like domain-containing protein | UT13 | Proteolysis |
| CSUI_005579 | 1.55 | 6,15E+09 | ubiquitin carboxyl-terminal hydrolase uchl3 | UT23_UT13 | Proteolysis |
| CSUI_009239 | 1.13 | 7,31E+08 | t1 family protein | UT13 | Proteolysis |
| CSUI_000247 | 1.11 | 6,39E+04 | subtilisin sub8 | UT13 | Proteolysis |
| CSUI_001067 | 4.38 | 5,32E+04 | subtilisin sub7 | UT12_UT23_UT13 | Proteolysis |
| CSUI_003817 | 1.93 | 2,01E+07 | subtilisin sub5 | UT13 | Proteolysis |
| CSUI_005044 | 2.40 | 8,77E+08 | subtilisin sub4 | UT12_UT13 | Proteolysis |
| CSUI_007469 | 3.57 | 2,54E+04 | subtilisin sub3 | UT12_UT13 | Proteolysis |
| CSUI_003180 | 1.03 | 3,61E+08 | sac3 ganp family protein | UT13 | Proteolysis |
| CSUI_007688 | 1.06 | 4,31E+09 | ring box protein 1 family protein | UT13 | Proteolysis |
| CSUI_000401 | 1.27 | 8,65E+08 | proteasome non-atpase 26s subunit | UT13 | Proteolysis |
| CSUI_003560 | 2.70 | 4,78E+04 | peptidase family m3 protein | UT12_UT23_UT13 | Proteolysis |
| CSUI_003171 | 1.00 | 0.028103 | peptidase family c50 protein | UT12 | Proteolysis |
| CSUI_006031 | 1.29 | 2,24E+06 | peptidase c13 family protein | UT13 | Proteolysis |
| CSUI_010687 | 2.23 | 4,39E+07 | pan domain-containing protein | UT12_UT13 | Proteolysis |
| CSUI_004468 | 2.65 | 1,07E+07 | pan domain protein | UT12_UT13 | Proteolysis |
| CSUI_006575 | 1.06 | 6,11E+03 | MINDY_DUB domain-containing protein | UT12 | Proteolysis |
| CSUI_004003 | 2.10 | 1,35E+07 | microneme protein mic15 | UT23_UT13 | Proteolysis |
| CSUI_003616 | 2.74 | 2,60E+06 | microneme protein | UT12_UT13 | Proteolysis |
| CSUI_002361 | 2.01 | 1,18E+07 | microneme protein | UT12_UT13 | Proteolysis |
| CSUI_004364 | 1.51 | 7,97E+09 | microneme protein | UT13 | Proteolysis |
| CSUI_007432 | 1.34 | 1,13E+04 | hypothetical protein-APPLE-PAN domain | UT13 | Proteolysis |
| CSUI_000729 | 1.80 | 2,50E+06 | hypothetical protein | UT12_UT13 | Proteolysis |
| CSUI_004556 | 1.17 | 0.018992 | hypothetical protein | UT12 | Proteolysis |
| CSUI_000719 | 4.36 | 4,91E+04 | hydrolase family protein | UT12_UT23_UT13 | Proteolysis |
| CSUI_002616 | 1.28 | 0.040510 | hect-domain (ubiquitin-transferase) domain-containing | UT12 | Proteolysis |
| CSUI_000223 | 1.40 | 9,57E+08 | heat shock protein hslv | UT13 | Proteolysis |
| CSUI_000444 | 1.47 | 1,62E+09 | e3 ubiquitin-protein ligase sinat2-like | UT13 | Proteolysis |
| CSUI_001155 | 2.32 | 1,38E+07 | Cullin_Nedd8 domain-containing protein | UT23_UT13 | Proteolysis |
| CSUI_001287 | 1.82 | 6,92E+04 | caax protease self-immunity protein | UT12_UT13 | Proteolysis |
| CSUI_000409 | 2.10 | 6,54E+08 | autophagy-related protein 8 atg8 | UT23_UT13 | Proteolysis |
| CSUI_004036 | 1.08 | 1,63E+04 | aminopeptidase n | UT23_UT13 | Proteolysis |
| CSUI_002080 | 1.14 | 7,60E+07 | 26s proteasome subunit p55 | UT23 | Proteolysis |
| CSUI_004236 | 1.07 | 1,45E+09 | 26s proteasome regulatory subunit 7 | UT23 | Proteolysis |
|  |  |  |  |  |  |
| CSUI_006690 | 1.91 | 3,77E+06 | aldo keto reductase family oxidoreductase | UT23_UT13 | Oxidoreductase |
| CSUI_010413 | 1.20 | 8,93E+08 | apicoplast-associated thioredoxin family protein | UT13 | Oxidoreductase |
| CSUI_000899 | 2.65 | 2,50E+05 | aromatic amino acid hydrolase | UT23_UT13 | Oxidoreductase |
| CSUI_005726 | 2.02 | 9,42E+05 | endoplasmic reticulum oxidoreductin | UT23_UT13 | Oxidoreductase |
| CSUI_008593 | 1.31 | 7,12E+04 | erv1 alr family protein | UT13 | Oxidoreductase |
| CSUI_003497 | 1.45 | 5,90E+06 | glutamate-5-semialdehyde dehydrogenase | UT13 | Oxidoreductase |
| CSUI_000027 | 1.02 | 0.015827 | glutaredoxin domain-containing protein | UT13 | Oxidoreductase |
| CSUI_003666 | 1.06 | 4,38E+05 | glyceraldehyde-3-phosphate dehydrogenase | UT13 | Oxidoreductase |
| CSUI_002609 | 3.43 | 1,75E+05 | hypothetical protein | UT12_UT23_UT13 | Oxidoreductase |
| CSUI_000729 | 1.80 | 2,50E+06 | hypothetical protein | UT12_UT13 | Oxidoreductase |
| CSUI_003451 | 1.31 | 1,21E+06 | lactate dehydrogenase ldh1 | UT13 | Oxidoreductase |
| CSUI_001724 | 2.41 | 5,99E+08 | mam domain-containing protein | UT12_UT13 | Oxidoreductase |
| CSUI_000414 | 2.47 | 9,93E+05 | mandelonitrile lyase | UT12_UT13 | Oxidoreductase |
| CSUI_000415 | 2.42 | 4,42E+07 | mandelonitrile lyase | UT12_UT13 | Oxidoreductase |
| CSUI_004727 | 2.66 | 6,48E+08 | nad transhydrogenase subunit beta | UT12_UT13 | Oxidoreductase |
| CSUI_000421 | 2.22 | 4,42E+09 | Oxidoreductase | UT12_UT13 | Oxidoreductase |
| CSUI_001721 | 1.13 | 3,88E+08 | peroxiredoxin | UT13 | Oxidoreductase |
| CSUI_008814 | 1.28 | 6,29E+05 | peroxiredoxin prx3 | UT13 | Oxidoreductase |
| CSUI_000575 | 3.25 | 8,06E+03 | protein disulfide-isomerase domain | UT12_UT13 | Oxidoreductase |
| CSUI_011096 | 2.44 | 3,72E+04 | ribonuclease h1 h2 small subunit protein | UT12_UT23_UT13 | Oxidoreductase |
| CSUI_005724 | 3.23 | 4,58E+04 | ribonucleoside-diphosphate reductase large subunit | UT12_UT23_UT13 | Oxidoreductase |
| CSUI_002110 | 1.09 | 1,90E+03 | ribonucleoside-diphosphate reductase large subunit | UT23_UT13 | Oxidoreductase |
| CSUI_005829 | 1.02 | 6,26E+07 | ribonucleoside-diphosphate reductase small subunit | UT13 | Oxidoreductase |
| CSUI_000109 | 1.14 | 3,88E+07 | succinate-semialdehyde dehydrogenase | UT13 | Oxidoreductase |
| CSUI_004134 | 1.24 | 3,33E+07 | thioredoxin | UT13 | Oxidoreductase |
| CSUI_002181 | 1.01 | 1,17E+09 | thioredoxin domain-containing protein | UT13 | Oxidoreductase |
| CSUI_007568 | 1.62 | 2,02E+07 | thioredoxin domain-containing protein | UT13 | Oxidoreductase |
| CSUI_006378 | 1.96 | 7,31E+08 | thioredoxin domain-containing protein | UT23_UT13 | Oxidoreductase |
| CSUI_009789 | 1.13 | 2,49E+04 | thioredoxin family redox-active protein | UT13 | Oxidoreductase |
| CSUI_010730 | 1.43 | 7,32E+04 | thioredoxin reductase | UT23_UT13 | Oxidoreductase |
| CSUI_011078 | 1.01 | 5,67E+03 | zinc finger cdgsh type protein | UT13 | Oxidoreductase |
| CSUI_001762 | 3.83 | 3,83E+01 | short chain dehydrogenase reductase family protein | UT12_UT13 | Oxidoreductase |
| CSUI_002755 | 1.30 | 6,36E+04 | adrenodoxin-type ferredoxin | UT13 | Oxidoreductase |
| CSUI_004068 | 1.09 | 1,61E+09 | carbonyl reductase | UT13 | Oxidoreductase |
| CSUI_005786 | 4.19 | 1,39E+06 | fad-dependent Oxidoreductase | UT12_UT23_UT13 | Oxidoreductase |
| CSUI_011295 | 1.20 | 3,32E+04 | 3-ketoacyl- reductase | UT23 | Oxidoreductase |
| CSUI_003444 | 1.43 | 3,87E+07 | sterol carrier protein-2 had-2scp-2 | UT13 | Oxidoreductase |
|  |  |  |  |  |  |
| CSUI_010330 | 1.42 | 4,05E+05 | acyl carrier protein | UT13 | Fatty acid met |
| CSUI_002440 | 2.75 | 2,23E+06 | acyltransferase domain-containing protein | UT12_UT23_UT13 | Fatty acid met |
| CSUI_001625 | 1.38 | 2,82E+07 | acyltransferase domain-containing protein | UT13 | Fatty acid met |
| CSUI_001485 | 1.17 | 7,77E+08 | beta-hydroxyacyl-acyl carrier protein dehydratase | UT13 | Fatty acid met |
| CSUI_006927 | 1.31 | 3,18E+08 | beta-ketoacyl-acyl carrier protein synthase iii | UT13 | Fatty acid met |
| CSUI_004552 | 1.74 | 7,00E+05 | fatty acid elongase | UT23_UT13 | Fatty acid met |
| CSUI_001418 | 2.87 | 5,94E+04 | Lipase_3 domain-containing protein | UT12_UT23_UT13 | Fatty acid met |
| CSUI_008540 | 1.02 | 1,09E+09 | type i fatty acid synthase | UT12 | Fatty acid met |
| CSUI_003072 | 1.07 | 1,32E+06 | triacylglycerol lipase | UT13 | Fatty acid met |
| CSUI_005878 | 1.21 | 1,10E+04 | cdp-alcohol phosphatidyltransferase sUerfamily protein | UT13 | Fatty acid met |
| CSUI_002551 | 1.07 | 0.017852 | cdp-alcohol phosphatidyltransferase sUerfamily protein | UT13 | Fatty acid met |
| CSUI_003527 | 1.61 | 5,40E+07 | cdp-diacylglycerol--inositol 3-phosphatidyltransferase 1-like | UT12_UT13 | Fatty acid met |
| CSUI_008766 | 1.32 | 2,46E+04 | dhhc zinc finger domain-containing protein | UT13 | Fatty acid met |
| CSUI_004715 | 1.01 | 4,29E+03 | dhhc zinc finger domain-containing protein | UT13 | Fatty acid met |
| CSUI_009793 | 1.75 | 1,89E+07 | glycerophosphodiester phosphodiesterase family protein | UT23_UT13 | Fatty acid met |
| CSUI_007585 | 1.33 | 1,11E+07 | phospholipase | UT23_UT13 | Fatty acid met |
| CSUI_003166 | 1.24 | 7,96E+04 | rft protein | UT13 | Fatty acid met |
| CSUI_007131 | 1.43 | 1,69E+09 | start domain protein | UT13 | Fatty acid met |
| CSUI_009671 | 2.85 | 5,37E+05 | start domain-containing protein | UT12_UT23_UT13 | Fatty acid met |
|  |  |  |  |  |  |
| CSUI_007070 | 4.06 | 2,69E+03 | fasciclin domain protein | UT12_UT23_UT13 | Surface |
| CSUI_006459 | 4.41 | 3,07E+04 | fasciclin domain-containing protein | UT12_UT23_UT13 | Surface |
| CSUI_002476 | 1.08 | 4,52E+09 | outer omp85 family protein | UT13 | Surface |
| CSUI_006179 | 3.96 | 3,97E+01 | SAG domain-containing protein | UT12_UT23_UT13 | Surface |
| CSUI_004248 | 3.06 | 1,29E+06 | sag-related sequence srs26i | UT12_UT23_UT13 | Surface |
| CSUI_005667 | 1.57 | 3,74E+08 | sag-related sequence srs28 | UT23_UT13 | Surface |
| CSUI_009850 | 3.09 | 6,33E+08 | proteophosphoglycan related | UpT12_UpT13 | Surface |
| CSUI_006635 | 1.68 | 7,28E+05 | proteophosphoglycan related | UpT23_UpT13 | Surface |
| CSUI_001278 | 2.85 | 3,81E+08 | proteophosphoglycan related protein | UpT12_UpT13 | Surface |
| CSUI_001693 | 1.11 | 1,10E+08 | longevity-assurance protein domain-containing protein | UT23 | Resistance |
| CSUI_001900 | 5.63 | 2,06E+03 | late embryogenesis abundant domain protein | UT12_UT23_UT13 | Resistance |
| CSUI_001899 | 5.31 | 1,95E+04 | late embryogenesis abundant domain protein | UT12_UT23_UT13 | Resistance |
| CSUI_005059 | 1.46 | 4,91E+09 | lea1 | UT13 | Resistance |

Uregulated transcripts coding for proteins with either a known or putative role in oocyst wall biosynthesis are listed along with their transcript abundance (LogFC), annotation and biological function.
