# Supplementary material for: The transcriptome from asexual to sexual in vitro development of Cystoisospora suis (Apicomplexa: Coccidia)
Source: Sci Rep. 2022 Apr 8;12:5972. doi: 10.1038/s41598-022-09714-8 (PMC8993856; doi:10.1038/s41598-022-09714-8)
Supplement: Supplementary file 10 — Supplementary Information 10. [file 41598_2022_9714_MOESM10_ESM.docx]

| Gene ID | logFC | FDR_adj_pval | Annotation | comparison | Function |
| --- | --- | --- | --- | --- | --- |
|  |  |  |  |  |  |
| CSUI_007002 | 1.23 | 5,46E+09 | centrin 2 | UT23_UT13 | Flagella |
| CSUI_006055 | 4.27 | 3,82E+03 | flagellar associated protein | UT12_UT23_UT13 | Flagella |
| CSUI_006910 | 2.80 | 3,12E+05 | flagellar associated protein | UT12_UT23_UT13 | Flagella |
|  |  |  |  |  |  |
| CSUI_003885 | 1.23 | 8,85E+08 | kinesin motor domain-containing protein | UT23_UT13 | microtubule movement |
| CSUI_005407 | 1.39 | 4,18E+09 | myosin a | UT13 | microtubule movement |
| CSUI_009311 | 1.96 | 2,06E+08 | myosin heavy chain | UT23_UT13 | microtubule movement |
| CSUI_000425 | 1.38 | 5,37E+03 | myosin k | UT13 | microtubule movement |
| CSUI_009385 | 1.18 | 2,96E+03 | myosin k | UT13 | microtubule movement |
| CSUI_005546 | 1.10 | 2,12E+09 | myosin light chain | UT13 | microtubule movement |
| CSUI_011403 | 1.20 | 5,16E+0 | myosin regulatory light chain | UT13 | microtubule movement |
| CSUI_000854 | 1.58 | 1,43E+07 | non-muscle myosin heavy | UT23_UT13 | microtubule movement |
| CSUI_007953 | 3.02 | 1,69E+05 | chromosome-associated kinesin klp1 | UT12_UT23_UT13 | microtubule movement |
|  |  |  |  |  |  |
| CSUI_007586 | 3.41 | 8,12E+06 | dynein gamma flagellar outer | UT12_UT23_UT13 | Axonema |
| CSUI_004717 | 1.62 | 7,86E+07 | dynein light chain dlc | UT23_UT13 | Axonema |
| CSUI_001333 | 1.08 | 1,22E+04 | dynein light chain roadblock-type 2 | UT13 | Axonema |
| CSUI_002604 | 2.73 | 7,88E+05 | growth arrest-specific protein 8 | UT12_UT23_UT13 | Axonema |
| CSUI_000245 | 2.21 | 4,06E+07 | heavy chain 2 family protein | UT23_UT13 | Axonema |
|  |  |  |  |  |  |
| CSUI_005725 | 1.08 | 2,04+03 | hypothetical protein | UT13 | MAPs |
| CSUI_000383 | 1.40 | 5,02E+04 | hypothetical protein | UT13 | MAPs |
| CSUI_004019 | 4.26 | 8,33E+04 | protein c21orf59 | UT12_UT23_UT13 | MAPs |
| CSUI_005284 | 1.81 | 9,50E+06 | septin | UT23_UT13 | MAPs |
| CSUI_006533 | 1.50 | 3,92E+09 | sf-assemblin beta giardin protein | UT23_UT13 | MAPs |
| CSUI_003799 | 1.00 | 4,28E+07 | sf-assemblin beta giardin protein | UT13 | MAPs |
| CSUI_008696 | 1.73 | 1,88E+04 | tubulin alpha chain | UT23_UT13 | MAPs |
| CSUI_009771 | 4.45 | 5,97E+05 | tubulin beta chain | UT12_UT23_UT13 | MAPs |
| CSUI_006267 | 1.99 | 5,80E+05 | tubulin beta chain | UT23_UT13 | MAPs |
| CSUI_006169 | 1.01 | 1,54E+05 | tubulin beta chain | UT13 | MAPs |
| CSUI_005420 | 1.18 | 5,17E+07 | beta-tubulin cofactor | UT23_UT13 | MAPs |
| CSUI_005608 | 1.58 | 4,02E+09 | tubulin binding cofactor a protein | UT23_UT13 | MAPs |
| CSUI_000829 | 1.03 | 1,82E+03 | tubulin-tyrosine ligase family protein | UT13 | MAPs |
| CSUI_000928 | 4.03 | 1,98E+05 | wd g-beta repeat-containing protein | UT12_UT23_UT13 | MAPs |
| CSUI_000362 | 3.43 | 3,09E+07 | wd g-beta repeat-containing protein | UT12_UT23_UT13 | MAPs |
| CSUI_007462 | 2.77 | 2,75E+07 | wd g-beta repeat-containing protein | UT12_UT23_UT13 | MAPs |
| CSUI_008538 | 1.28 | 3,38E+09 | wd g-beta repeat-containing protein | UT13 | MAPs |
| CSUI_007082 | 1.17 | 3,23E+08 | wd g-beta repeat-containing protein | UT13 | MAPs |
| CSUI_002527 | 1.10 | 2,13E+09 | wd g-beta repeat-containing protein | UT13 | MAPs |
| CSUI_006911 | 3.18 | 8,10E+04 | wd g-beta repeat-containing protein | UT12_UT23_UT13 | MAPs |
| CSUI_007648 | 1.32 | 3,04E+03 | wd g-beta repeat-containing protein | UT12_UT13 | MAPs |
| CSUI_005956 | 1.24 | 2,50E+05 | wd g-beta repeat-containing protein | UT13 | MAPs |
| CSUI_009641 | 1.01 | 4,97E+06 | wd-40 repeat | UT23 | MAPs |
| CSUI_005045 | 1.27 | 3,10E+07 | wd-40 repeat protein | UT13 | MAPs |
| CSUI_000729 | 1.80 | 2,50E+06 | hypothetical protein | UT12_UT13 | MAPs |
|  |  |  |  |  |  |
| CSUI_000472 | 3.19 | 2,40E+08 | male gamete fusion factor | UT12_UT23_UT13 | gamete fusion |
| CSUI_002998 | 3.20 | 1,95E+04 | morn repeat-containing protein | UT12_UT23_UT13 | cell budding |
| CSUI_000048 | 1.30 | 2,22E+07 | morn repeat-containing protein | UT13 | cell budding |
| CSUI_004816 | 1.70 | 2,78E+06 | morn repeat-containing protein | UT23_UT13 | cell budding |
|  |  |  |  |  |  |
