# Supplementary material for: The transcriptome from asexual to sexual in vitro development of Cystoisospora suis (Apicomplexa: Coccidia)
Source: Sci Rep. 2022 Apr 8;12:5972. doi: 10.1038/s41598-022-09714-8 (PMC8993856; doi:10.1038/s41598-022-09714-8)
Supplement: Supplementary file 11 — Supplementary Information 11. [file 41598_2022_9714_MOESM11_ESM.docx]

**Table 5**. Primers used in this work

| N | Primer | Sequences (5’→ 3’) | Amplicon length (bp) |  |  |
| --- | --- | --- | --- | --- | --- |
|  |  |  |  |  |  |
| 1 | Fw-ACTIN | CTTGCTGGCCGTGATTTGAC |  |  |  |
| 2 | Rv-ACTIN | ATATTGCCGTCCGGAAGCTC | 203 |  |  |
| 3 | Probe-ACTIN | CCTCCGCCGAGAAGGAAATT |  |  |  |
|  |  |  |  |  |  |
| 4 | Fw-GAPDH | TTCAACGAGAAGGAGCCAAG |  |  |  |
| 5 | Rv-GAPDH | CTTCGGAGGTGCAGACATG | 150 |  |  |
| 6 | Probe-GAPDH | CAAGGAAAAGGCTGAGGCGCAT |  |  |  |
|  |  |  |  |  |  |
| 7 | Fw- CSUI_008252 | GTGGCATCCTCAAGGAAGAA |  |  |  |
| 8 | Rv- CSUI_008252 | GGAACAGAGGTTCCGAGTATG | 155 |  |  |
| 9 | Probe- CSUI_008252 | CGCATACGGACCCTAGTTGTGCTC |  |  |  |
|  |  |  |  |  |  |
| 10 | Fw- CSUI_003709 | GGAGAACGACGTAGTTAACATGA |  |  |  |
| 11 | Rv- CSUI_003709 | GCACCAAGGTCTTCACAATAATC | 137 |  |  |
| 12 | Probe- CSUI_003709 | TGCTGCGCGTGACTGTATAATCCA |  |  |  |
|  |  |  |  |  |  |
| 13 | Fw- CSUI_000190 | CATCTGTGCAGTCGGAAGAG |  |  |  |
| 14 | Rv- CSUI_000190 | ATAAGGTACGGGAGCGTAGT | 123 |  |  |
| 15 | Probe- CSUI_000190 | TATGGAGGCCCAGTCTTCTACGGT |  |  |  |
|  |  |  |  |  |  |
| 16 | Fw- CSUI_005927 | GTCCATGCTGAGGGTCTTATAC |  |  |  |
| 17 | Rv- CSUI_005927 | CTATGGTGTTGCTCCTCTCTTT | 177 |  |  |
| 18 | Probe- CSUI_005927 | ACGTGAAGGCCGAGAACTTTGTCT |  |  |  |
|  |  |  |  |  |  |
| 19 | Fw- CSUI_003422 | GAGGAAGGCACTGATCAAAGAG |  |  |  |
| 20 | Rv- CSUI_003422 | TATCCGAACCGCCAGAGT | 150 |  |  |
| 21 | Probe- CSUI_003422 | AGAAGCTAGACAAGCAGCAGGGTT |  |  |  |
|  |  |  |  |  |  |
| 22 | Fw- CSUI_006265 | CGTGCTTAGTCCTCCCAAAT |  |  |  |
| 23 | Rv- CSUI_006265 | CACATCGTGACCATACTCTCTG | 169 |  |  |
| 24 | Probe- CSUI_006265 | CAACAAAGACACCAGGCGCTGTTT |  |  |  |
|  |  |  |  |  |  |
| 25 | Fw- CsTyRP-exp | CACCATGGAGGCTCTACGTTTTTCCATCG | 1062 |  |  |
| 26 | Rv- CsTyRP-exp | TTAATAACAACCTGTGCCTTTTGAGCAGT |  |  |  |
|  |  |  |  |  |  |
|  |  |  |  |  |  |
